# The Post-transcriptional Regulator *rsmA*/*csrA* Activates T3SS by Stabilizing the 5′ UTR of *hrpG*, the Master Regulator of *hrp/hrc* Genes, in *Xanthomonas*

**DOI:** 10.1371/journal.ppat.1003945

**Published:** 2014-02-27

**Authors:** Maxuel O. Andrade, Chuck S. Farah, Nian Wang

**Affiliations:** 1 Citrus Research and Education Center, Department of Microbiology and Cell Sciences, University of Florida, Lake Alfred, Florida, United States of America; 2 Department of Biochemistry, Institute of Chemistry, University of São Paulo, São Paulo, São Paulo, Brazil; University of California Riverside, United States of America

## Abstract

The RsmA/CsrA family of the post-transcriptional regulators of bacteria is involved in the regulation of many cellular processes, including pathogenesis. In this study, we demonstrated that *rsmA* not only is required for the full virulence of the phytopathogenic bacterium *Xanthomonas citri* subsp. citri (XCC) but also contributes to triggering the hypersensitive response (HR) in non-host plants. Deletion of *rsmA* resulted in significantly reduced virulence in the host plant sweet orange and a delayed and weakened HR in the non-host plant *Nicotiana benthamiana*. Microarray, quantitative reverse-transcription PCR, western-blotting, and GUS assays indicated that RsmA regulates the expression of the type 3 secretion system (T3SS) at both transcriptional and post-transcriptional levels. The regulation of T3SS by RsmA is a universal phenomenon in T3SS-containing bacteria, but the specific mechanism seems to depend on the interaction between a particular bacterium and its hosts. For Xanthomonads, the mechanism by which RsmA activates T3SS remains unknown. Here, we show that RsmA activates the expression of T3SS-encoding *hrp/hrc* genes by directly binding to the 5′ untranslated region (UTR) of *hrpG*, the master regulator of the *hrp/hrc* genes in XCC. RsmA stabilizes *hrpG* mRNA, leading to increased accumulation of HrpG proteins and subsequently, the activation of *hrp/hrc* genes. The activation of the *hrp/hrc* genes by RsmA via HrpG was further supported by the observation that ectopic overexpression of *hrpG* in an *rsmA* mutant restored its ability to cause disease in host plants and trigger HR in non-host plants. RsmA also stabilizes the transcripts of another T3SS-associated *hrpD* operon by directly binding to the 5′ UTR region. Taken together, these data revealed that RsmA primarily activates T3SS by acting as a positive regulator of *hrpG* and that this regulation is critical to the pathogenicity of XCC.

## Author Summary

Pathogenic bacteria demonstrate sophisticated capacity to regulate gene expression to meet requirements of living in different environmental niches, including in the hosts. The activation of the Type 3 secretion system (T3SS) genes in response to the host enviroment is under the control of several factors, such as the post-transcriptional regulator RsmA/CsrA. Here, we show that RsmA contributes to the pathogenicity of *Xanthomonas citri* in host plants and the HR-triggering activity in non-host plants by regulating the expression of T3SS-encoding *hrp/hrc* genes. RsmA directly interacts with the 5′ UTRs of *hrpG* and *hrpD* mRNAs, which leads to increased HrpG protein levels by stabilizing the *hrpG* transcript. Further, overexpression of *hrpG* in an *rsmA* mutant restored its pathogenicity and ability to cause HR. The deletion of *rsmA* did not affect the phosphorylation of HrpG, which is also required for T3SS activation. This work provides mechanistic insights for the first time into RsmA-mediated regulation of T3SS gene expression by acting as a positive regulator of *hrpG* at the post-transcription level.

## Introduction

Pathogenic bacteria belonging to the genus *Xanthomonas* cause diseases in many economically important plants throughout the world. The virulence of these bacteria depends on a type 3 protein secretion system (T3SS) [1], [2], [3]. T3SS mediates the translocation of bacterial effector proteins into the host cell, where they may interfere with host metabolic pathways and/or suppress plant defense reactions [4], [5], [6], [7].Genes encoding the T3SS are referred to as Hypersensitive Response and Pathogenicity (*hrp*) genes because *hrp* mutants lost the abilities to trigger the HR in non-host plants and pathogenesis in host plants [8]. In Xanthomonads, the *hrp* gene cluster contains at least 22 genes, nine of which are highly conserved and therefore have been termed *hrc* (*hrp* conserved) genes [4]. The expression of the *hrp* cluster of genes in *Xanthomonas* has been shown to be activated *in planta* and in the minimal medium XVM2 by the transcriptional regulators HrpG and HrpX [9], [10]. HrpG is a response regulator belonging to the OmpR family of two-component system response regulators that contains an N-terminal response receiver (RR) domain and a C-terminal DNA-binding motif [10], [11]. Recently, a putative cognate sensor histidine kinase was reported for HrpG in *Xanthomonas campestris*, but further studies are needed to identify the activation signal and whether homologs of this sensor kinase are functional in other *Xanthomonas* species [1], [7], [12]. Phosphorylated HrpG is predicted to activate the expression of *hrpX*, which encodes an AraC-type regulator [11]. HrpX binds to a *cis*-regulatory element, the plant inducible promoter (PIP) conserved motif (TTCGC-N_15_-TTCGC) or a less-conserved PIP-like motif (TTCGC-N_8_-TTCGT), which is present in the promoter regions of four operons (*hrpB*, *hrpC*, *hrpD* and *hrpE*), *hrpF* within the *hrp* cluster (Fig. S1), and a set of genes involved in the virulence of Xanthomonads [4], [9], [13], [14]. Likewise, HrpG and HrpX homologs have been reported to mediate the expression of genes in the *hrp* cluster of the plant pathogenic bacteria *Ralstonia solanacearum* and the animal pathogen *Burkholderia pseudomallei* [15], [16]. The trigger signal and post-transcriptional regulation of HrpG/HrpX-dependent signaling pathway, which is different from that in *Erwinia carotovora* and *Pseudomonas* spp., denominated type I regulatory system, are poorly understood [7], [17], [18].

Further studies in Xanthomonads indicate that the post-transcriptional regulator RsmA (repressor of secondary metabolism) positively regulates pathogenicity [19], [20]. RsmA belongs to a conserved family of RNA-binding proteins that were initially identified as repressors of carbon metabolism (carbon storage regulator [CsrA]) [21], [22]. Members of the RsmA/CsrA family are widely distributed among eubacteria and control various traits including biofilm formation, motility, carbon flux, secondary metabolism, quorum-sensing, and virulence to animal and plant hosts [22], [23], [24], [25], [26], [27], [28]. RsmA/CsrA has been shown to regulate the T3SS genes of many animal and plant pathogenic bacteria including *Pseudomonas aeruginosa* [29], *Salmonella enterica* Serovar Typhimurium [28], *Erwinia carotovora* [30], [31], *Dickeya dadantii* [32], and *Xanthomonas* spp. [19], [20]. In *X. campestris* pv. campestris and *X. oryzae* pv. oryzae, deletion of an *rsmA* homolog resulted in a complete loss of virulence in host and a loss of HR induction in non-host plants, impairment of endoglucanase production, and enhanced biofilm formation [19], [20]. While T3SS genes have been shown to be regulated by RsmA homologs in many animal and plant pathogenic bacteria, the mechanistic understanding of RsmA-mediated regulation of T3SS genes in Xanthomonads remained unknown.

RsmA can bind to the 5′ untranslated regions (UTRs) of specific mRNAs by recognizing a wide variety of sequences containing the conserved trinucleotide motif GGA in the loop structures [22], [33], [34], [35]. By directly binding to the target mRNAs, RsmA can either down- or up-regulate the expression of target genes or inhibit translation by blocking the access of the ribosome to the Shine-Dalgarno sequence of the specific mRNA [25], [36], [37]. In animal and plant pathogenic bacteria including *Escherichia coli*, *S. enterica* Serovar Typhimurium, *P. aeruginosa*, and *E. carotovora*, RsmA/CsrA proteins are inhibited by small noncoding RNAs (*rsmB* family) with various GGA motifs present in the loops of the secondary structure [25], [38], [39], [40], [41]. The *rsmB* RNA can bind and sequester multiple copies of RsmA/CsrA [25], [42]. However, *rsmB* homologs have not been identified in *Xanthomonas* spp. genomes, suggesting that RsmA regulation involves a novel, as-yet-undiscovered mechanism.

Despite that RsmA/CsrA represses the translation initiation of a variety of genes, a molecular mechanism by which RsmA/CsrA mediates the activation of the *flhDC* operon, which encodes the master regulator of flagellum synthesis, has recently been identified in *E. coli* [43], [44]. RsmA/CsrA binds to two sites of the *flhDC* leader sequence, and by interfering with the 5′ end-dependent RNAse E cleavage pathway, RsmA/CsrA can stabilize the *flhDC* transcript [44].

To advance the mechanistic understanding of the RsmA regulation of T3SS genes in Xanthomonads, we investigated how RsmA activates the *hrp/hrc* genes of *Xanthomonas citri* subsp. citri (XCC), which causes the important citrus canker disease throughout the world [45]. In this paper, we present a model in which RsmA positively regulates the *hrp/hrc* genes in *Xanthomonas*, and discuss the implications of these findings in plant host-pathogen interactions.

## Results

### RsmA plays a central role in the activation of the pathogenicity and HR of XCC

The *rsmA* gene of XCC (XAC1743, GenBank accession number NP_642074) consists of 213 bp nucleotides and encodes a protein comprising 70 amino acids [46]. RsmA of XCC displays 74% identity with *E. coli* K12 CsrA (NP_417176.1), 93% with *X. campestris* pv. campestris strain 8004 RsmA (AAY49556), and 100% with *X. oryzae* pv. *oryzae* strain 13751 XOO_2760 (NC_007705.1).

The sequence alignment of *Xanthomonas* RsmA with homologous proteins exhibited many highly conserved residues in its primary structure [19]. One notable exception is the presence of nine additional residues in the carboxy-terminal region of RsmA of *Xanthomonas* spp. that do not exist in RsmA homologs in Enterobacteria, *Pseudomonas* spp. and *Erwinia* spp. (Fig. S2A) [30], [47], [48]. An unusual C-terminal extension is also present in an RsmA homolog in *Sinorhizobium meliloti* (RsmAsm) which is 81% identical to XCC RsmA [49]. Deletion of the extended C-terminal of RsmAsm seems to affect its cellular concentration, but enhances its relative RNA binding activity. Interestingly, computational analysis of the predicted secondary structure of XCC RsmA by Phyre [50] revealed that a unique α-helix motif is formed between residues 44–60 (Fig. S2A), in contrast to RsmA homologs from other species with α-helix motifs restricted to residues 44–53 [33], [34]. This difference could be caused by the extended C-terminal sequence in the *Xanthomonas* RsmA. Furthermore, comprehensive alanine-scanning mutagenesis and structural studies of RsmA homologs identified the residues Leu2, Leu4, Arg6, Arg7, Val40, Val42, Arg44 and Ile47, but not the motif GxxG as hypothesized in previous reports [33], [34], [48], [51], as required for interactions with mRNA targets [33], [34]. We analyzed the three-dimensional structure model of XCC RsmA using the Swiss-Model Repository program [52], [53], [54] (http://swissmodel.expasy.org) with *E. coli* CsrA structure as a model (PDB: 1Y00, [48]), and visualized it in Pymol [55] (Fig. S2B). As the determinate structures of RsmA homologs in *E. coli*, *P. aeruginosa* and *Yersinia enterocolitica* (PDBs: 1Y00, 1VPZ and 2BTI, respectively), the structural model of XCC RsmA is a homodimer with identical RNA-binding surfaces that can simultaneously bind to two sites within a transcript. The critical RsmA/CsrA residues involved in RNA-binding are conserved in the *Xanthomonas* RsmA and highlighted in the XCC RsmA structure model (Fig. S2).

**Figure 1:**
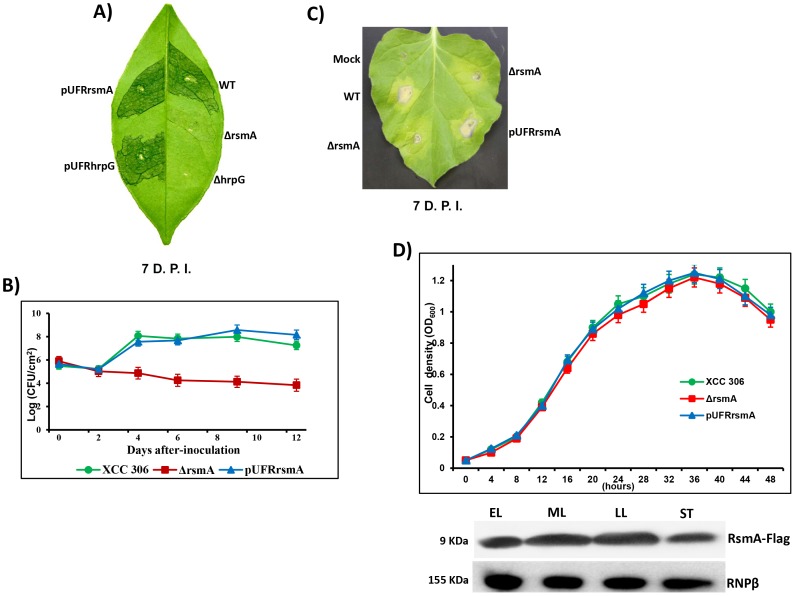
*rsmA* is required for the pathogenicity of *Xanthomonas citri* subsp. citri in the host plant sweet orange and contributes to the hypersensitive response (HR) in tobacco leaves (*Nicotiana benthamiana*). **A**) Disease symptoms on host sweet orange (*Citrus sinensis*) leaves 7 days post inoculation (D.P.I.) of bacterial cells at a concentration of 10^6^ CFU/ml. **B**) Growth assay in planta. Bacterial cells were inoculated into sweet orange leaves at a concentration of 10^6^ CFU/ml and recovered at different time points. The values represent the means of three replicates. The experiment was repeated three times with similar results. Means ± standard deviations are plotted. **C**) Macroscopic symptoms induced 7 D.A.I of tobacco leaves by infiltrating bacterial cells at a concentration of 10^6^ CFU/ml. **D**) Growth curve in the minimal medium XVM2 and Western-blotting assay using protein extracts of *rsmA* mutant cells harboring the pUFR047-*rsmA*-Flag construct. Cells were collected in different growth stages: EL, early log; ML, medium log); LL, late log; and ST, stationary phase. Wt = *X. citri* subsp. *citri* strain 306, Δrsm*A* = mutant with a deletion of XAC1743 (*rsmA*) harboring the empty plasmid pUFR047, pUFRrsmA = complementation of ΔrsmA with *rsmA* cloned in pUFR047, ΔhrpG = *hprG* mutant [9], pUFRhrpG = complementation of ΔhrpG with *hrpG* cloned into pUFR047, and Mock, 10 mM MgCl2. RNPβ: antibody to the β-subunit of RNA polymerase.

To study the contribution of *rsmA* to the pathogenicity of XCC, we employed an allelic exchange protocol to construct the Δ*rsmA* mutant of XCC strain 306 containing an in-frame deletion of *rsmA* codons 5–66 (Table S1). The deletion of *rsmA* was confirmed by PCR, and the complementation with wild-type *rsmA* restored the virulence in the host plants (Fig. 1).

While infection of the host plant sweet orange (*Citrus sinensis*) with wild-type XCC strain resulted in clear canker disease symptoms, the Δ*rsmA* strain caused less hypertrophy and hyperplasia and failed to produce water-soaking and necrosis symptoms in the susceptible citrus host (Fig. 1A). Consistent with the reduced symptom development caused by the *rsmA* mutant, our results also indicated that *rsmA* is essential for the proliferation of XCC in host plants (Fig. 1B). The deletion of *rsmA* impaired the growth of XCC inside the host, whereas the complemented strain multiplied to the same level as wild-type (Fig. 1B). These results are consistent with previous studies showing the requirement of *rsmA* homologs for the virulence of *X. campestris* pv. campestris and *X. oryzae* pv. oryzae in their specific hosts [19], [20]. In addition, it was observed that *rsmA* contributes to HR induction in the non-host tobacco plant (*Nicotiana benthamiana*) (Fig. 1C).

To determine whether mutation in *rsmA* has any effect on the growth of XCC in media, we examined the growth of the *rsmA* mutant strain (Δ*rsmA*), the wild-type strain XCC and the complemented strain pUFRrsmA in the minimal medium XVM2 [56], [57]. The results showed that the Δ*rsmA* and wild-type XCC strains grew to similar levels in the XVM2 medium (Fig. 1D), indicating that *rsmA* is not required for the growth of XCC in the XVM2 minimal medium. We also observed an increase in RsmA protein levels in the mid-log and late-log growth stages of wild-type XCC carrying the pUFRrsmA-flag construct (Fig. 1D).

### RsmA positively regulates the expression of *hrp/hrc* genes

The significant contribution of RsmA to the pathogenicity and HR-triggering activity of XCC prompted us to investigate its regulatory effect on the *hrp/hrc* genes by confirming whether RsmA controls the expression of T3SS genes as has been observed in other pathogenic bacteria. The expression of *hrp/hrc* genes in Xanthomonads has been reported to be repressed in nutrient-rich media but strongly induced *in planta* and in the XVM2 medium [10], [56]. A previous microarray analysis showed that the whole XCC *hrp* gene cluster, which contains 24 genes (XAC0393 to XAC0417) (Fig. S1), was down-regulated in *hrpG* and *hrpX* mutants of XCC grown in XVM2 medium [9].

To test whether RsmA regulates the expression of *hrp/hrc* genes in XCC, microarray analyses were conducted to compare the gene expression of the Δ*rsmA* mutant with that of wild-type XCC strain 306 grown in XVM2 medium to OD_600 nm_ = 0.5. In this study, false discovery rate (FDR) = 0.05 and absolute value of log_2_-fold change = 1 (equivalent to a fold change of 2) were used as the cutoff values. Compared to wild-type XCC strain 306, a total of 82 genes were down-regulated and 88 genes were up-regulated in the Δ*rsmA* mutant (Table S2). Overall, 23 genes in the *hrp* cluster and 16 genes encoding putative T3SS effectors were down-regulated in the Δ*rsmA* mutant (Table S2). The microarray data were validated by quantitative reverse-transcription PCR (qRT-PCR) of 11 *hrp* genes. Although we observed differences in the amplitude of fold changes determined using the two techniques, the general trends in gene expression were consistent for all 11 genes that were tested (Table 1).

**Table 1:** *rsmA* regulation of the expression of the genes in the *hrp* cluster of *Xanthomonas citri* subsp. citri.

Locus tag	Gene name	Log_2_ ratio of fold change (ΔrsmA/WT)^a^	Log_2_ ratio of fold change (pBRArsmA/pBRA-XCC)^b^	qRT-PCR (ΔrsmA/WT)^a^	Product description
		Dowregulated	Upregulated		
XAC0394	*hrpF*	−1.23	2.79	-	HrpF
XAC0396	*hpaB*	−1.13	3.31	-	HpaB
XAC0397	*hrpE*	−1.44	2.65	−3.20	HrpE
XAC0398	*hrpD6*	−1.30	2.87	−3.04	HrpD6
XAC0399	*hrpD5*	−1.56	3.50	−2.76	HrpD5
XAC0400	*hpaA*	−1.25	3.58	-	HpaA
XAC0401	*hrcS*	−1.31	3.62	−2.94	HrcS
XAC0402	*hrcR*	−1.41	3.53	-	HrcR
XAC0403	*hrcQ*	−1.73	3.98	−3.32	HrcQ
XAC0404	*hpaP*	−1.10	3.37	-	HpaP
XAC0405	*hrcV*	−1.26	3.35	-	HrcV
XAC0406	*hrcU*	−1.40	3.78	−2.82	HrcU
XAC0407	*hrpB1*	−1.37	3.48	−3.14	HrpB1
XAC0408	*hrpB2*	−1.32	3.92	−2.65	HrpB2
XAC0409	*hrcJ*	−1.82	3.91	-	HrcJ
XAC0410	*hrpB4*	−1.75	4.05	-	HrpB4
XAC0411	*hrpB5*	−1.46	4.12	-	HrpB5
XAC0412	*hrcN*	−1.23	3.78	-	HrcN
XAC0413	*hrpB7*	−1.17	4.02	-	HrpB7
XAC0414	*hrcT*	−1.00	3.96	-	HrcT
XAC0415	*hrcC*	−1.06	2.51	−2.84	HrcC
XAC0416	*hpa1*	−1.72	3.28	−3.12	Hpa1
XAC0417	*hpa2*	−1.13	2.53	−2.48	Hpa2
XAC1265	*hrpG*	−1.42	2.02	−2.27	HrpG
XAC1266	*hrpX*	−1.38	2.11	−2.32	HrpX

a The Log_2_-fold change of each gene was derived by comparing the *rsmA* mutant versus wild-type (p<0.05; Log2> = 1).

b The Log_2_-fold change of each gene was derived by comparing the Δ*rsmA* mutant strains carrying the construct pBRArsmAxcc and the empty plasmid pBRA. Transcriptome analyses were performed with four independent biological samples for each condition.

### Induced overexpression of *rsmA* positively regulates *hrp/hrc* genes

To further investigate the regulation of *hrp/hrc* genes by RsmA, a recombinant construct named pBRA-*rsmA*, which contains *rsmA* fused to an arabinose-inducible promoter, was used to induce the overexpression of 6HisRsmA in the Δ*rsmA* mutant strain (Table 1). After induction with 0.3% L-arabinose in the XVM2 medium, the expression and functionality of 6HisRsmA were confirmed by Western-blotting and by measuring the extracellular endoglucanase activity (Fig. S3). The endoglucanase activity in the Δ*rsmA* mutant was restored to 67% of the wild-type level after the induction of 6HisRsmA (Fig. S3). Microarray analysis was also conducted to evaluate gene expression profiles in the Δ*rsmA* mutant bacterial cells transformed with pBRA-rsmA (strain pBRA-rsmA) or with the empty plasmid pBRA (strain pBRA-XCC). Again, the bacterial cells were cultured in the XVM2 medium supplemented with 0.3% L-arabinose at an OD_600 nm_ of 0.5. Using the same cutoff values, our microarray data showed that the overexpression of *rsmA* led to the activation of 72 genes and the down-regulation of 81 genes, corresponding to a 90% overlap with the differentially expressed genes identified in the Δ*rsmA*/WT microarray analysis (Table S2). The patterns of differential gene expression were inversely related between the the Δ*rsmA* mutant and pBRA-rsmA strains (Tables 1 and S2). The *hrp/hrc* genes exhibited the strongest activation after 6HisRsmA induction in the pBRA-rsmA strain (Table 1). While the deletion of *rsmA* resulted in the down-regulation of the *hrp/hrc* genes and significantly reduced virulence, the expression of T3SS genes was significantly activated by the overexpression of RsmA in the Δ*rsmA* mutant (Table 1). Taken together, these data confirmed that *rsmA* contributes to virulence by activating the expression of *hrp/hrc* genes.

### Deletion of *rsmA* reduces protein levels of T3SS components

**Figure 2:**
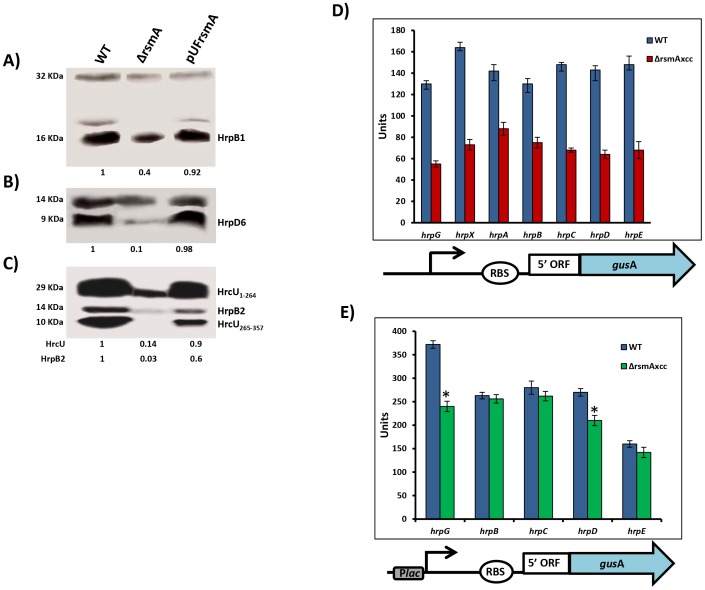
RsmA regulates protein levels of T3SS in *X. citri* subsp. citri. Immunoblotting analyses of the total protein extracts of the wild-type (Wt), the *rsmA* mutant (Δ*rsmA*) harboring the empty plasmid pUFR047 and the complemented strain (pUFRrsmA) are shown. Bacterial cells were grown in the XVM2 medium and collected at OD_600 nm_ = 0.5. Proteins were separated by SDS-PAGE and transferred to a nitrocellulose membrane. The blots were probed with **A**) HrpB1, **B**) HrpD6 or **C**) HrcU and HrpB2 polyclonal antibodies, respectively. Protein-A conjugated with horseradish peroxidase was used to detect the blots. Beneath the panels are presented the values of the relative levels of detected proteins in *rsmA* mutant and complemented strains which were estimated according to wild-type results. The estimated values were normalized with the values obtained to unspecific protein bands also recognized by the antibodies. **D**) and **E**) GUS assays using translational fusion constructs. The different constructs used in this assay are represented by diagrams bellow of the graphics. D) Wild-type and *rsmA* mutant cells harboring plasmid-borne promoterless *gusA* in-frame fused to the native promoters and the first codons of the *hrp* genes. E) Wild-type and *rsmA* mutant cells transformed with translational fusions driven by the constitutive P*lac* promoter. Values presented are means ± standard deviations of three independent experiments. * represents the significant difference between the wild-type and Δ*rsmA* values by using ANOVA. The GUS assay was repeated twice with similar results.

To investigate whether the deletion of *rsmA* also caused a reduction in the protein levels of T3SS, we performed Western-blotting experiments to evaluate the abundances of HrcU, HrpB1, HrpB2 and HrpD6 in total cell extracts of wild-type XCC strain 306, the Δ*rsmA* mutant, and the complementation strain grown in the XVM2 medium. Based on the immunobloting results, the relative protein levels in the Δ*rsmA* mutant was estimated in reference to the wild-type strain and normalized according to unspecific protein bands also recognized by the polyclonal antibodies (Figs. 2A, 2B and 2C). The protein levels of HrcU, HrpB1, HrpB2, and HrpD6 were significantly reduced to 14%, 40%, 3% and 10%, respectively, in the Δ*rsmA* mutant (Figs. 2A, 2B and 2C). Interestingly, the antibodies generated against HrcU recognized two peptides of approximately 28 and 10 KDa, respectively (Fig. 2C). Indeed, HrcU can undergo autocatalytic cleavage between the asparagine and the proline residues of a conserved NPTH motif (amino acids 264–267), resulting in a reorientation of the PTH loop, which is required for the secretion of late T3SS substrates [58], [59]. The relative protein levels of HrcU, HrpB1, HrpB2 and HrpD6 were restored by the expression of *rsmA* in the complemented strain (Figs. 2A, 2B and 2C).

The positive regulation of *hrp/hrc* genes by RsmA in XCC was further investigated using translational fusion constructs with a *gusA* reporter gene [60], [61]. The translational fusion constructs harbor the native promoter, the 5′ leader region and at least the first three codons of the coding DNA sequences of *hrpG*, *hrpX*, *hrpA* genes, as well as *hrpC*, *hrpD* and *hrpE* operons fused in-frame to the ORF (open reading frame) of *gusA* (Fig. 2D). To differentiate transcriptional and post-transcriptional regulation, we investigated whether replacing the original promoters of the *hrp/hrc* genes by constitutively expressed P*lac* promoter affects the GUS activities in wide type and the *rsmA* mutant strains (Fig. 2E). Results of the GUS activities demonstrated that the *rsmA* mutation significantly affected the expression of all *hrp* genes from their native promoters, but it specially affected the expression of *hrpG* and *hrpD* under the control of P*lac* (Figs. 2D, 2E). Taken together, these results indicated that RsmA regulates the expression of *hrp/hrc* genes at the transcriptional level, and may also control *hrpG* and *hrpD* expression at the post-transcriptional level.

### Mapping of the 5′-UTR sequences of *hrp/hrc* transcripts

Previous studies have suggested that RsmA binds to the consensus sequence, ACARGGAUG, with the GGA motif representing the most conserved nucleotides [35], [62]. RsmA can bind to the 5′-UTR of a specific mRNA and negatively or positively regulate the transcript stability and thus the translation [22]. Because RsmA activates all *hrp/hrc* genes including *hrpG* and *hrpX* (Table 1), the two master regulators of *hrp/hrc* genes, we hypothesized that RsmA exerts its regulation of the *hrp/hrc* genes via *hrpG*, the upmost in the regulatory hierarchy. Alternatively, RsmA could bind to the 5′ UTR of each individual transcription unit of the *hrp* cluster.

**Figure 3:**
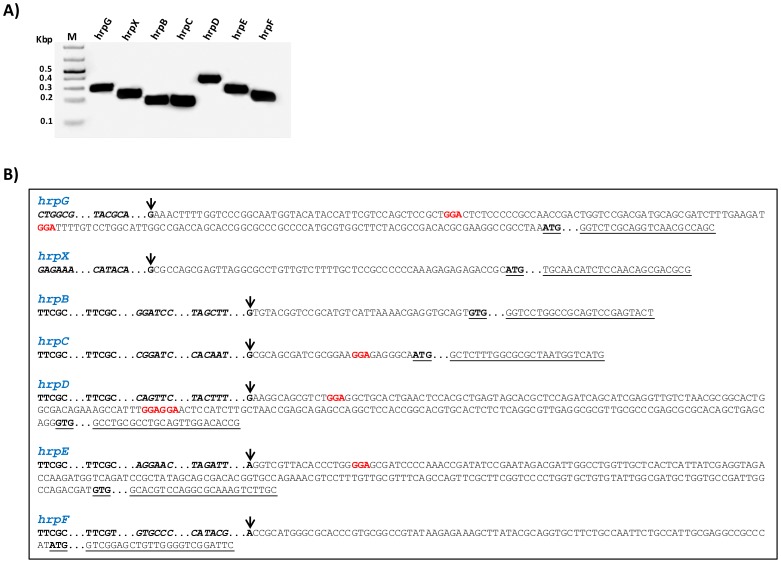
Determination of *hrp/hrc* transcriptional start sites of *X. citri* subsp. citri. The transcriptional start sites for *the genes hrpG, hrpX and hrpF, and the operons hrpB, hrpC, hrpD and hrpE* of XCC were determined by 5′RACE. **A**) Specific PCR products were detected after the amplification of reverse-transcribed cDNA with the gene-specific primers for *hrp/hrc genes* together with an adapter primer (Roche), respectively. **B**) Sequencing of the PCR products identified the nucleotides indicated by arrow as the transcription start sites of *hrpG, hrpX, hrpB, hrpC, hrpD, hrpE and hrpF* of XCC. Analysis of the 5′ leader sequences of the *hrp/hrc* transcripts suggests potential RsmA binding sites (highlighted) in *hrpG*, *hrpC*, *hrpD* and *hrpE*. However, the 5′ leader regions of the *hrpB*, *hrpF* and *hrpX* do not contain the GGA motifs. The +1 nucleotide is indicated with an arrow, putative RsmA binding sites (GGA) in the leader sequences are highlighted in red color, and the PIP-box motif within each promoter are in bold. The −35 and −10 sequences are shown in bold and italics. ATG or GTG are indicated in bold and underlined. The specific primers used to amplify the fragments are underlined.

The transcriptional initiation sites of the genes *hrpG*, *hrpX*, *hrpF*, and also of the operons *hrpB*, *hrpD*, *hrpD* and *hrpE* in XCC were determined by rapid amplification of 5′-cDNA Ends (5′ RACE) (Fig. 3A). The RACE PCR products were then sequenced to identify specific nucleotides, indicated by arrows in the Figure 3B, as the transcription start sites of individual transcription units. The analysis of the 5′ RACE results allowed us to determine the PIP-box promoters, -35 and -10 regions, leader sequences and the first codon for each *hrp/hrc* transcript in XCC (Fig. 3B).

The PIP-box promoters and leader sequences for the *hrp/hrc* transcripts were previously experimentally identified in *X. campestris* pv. vesicatoria and *X. oryzae* pv. oryzae [4], [13], [46], [63], [64]. Our results confirmed that the *hrp/hrc* transcriptional start sites identified in XCC are similar to those reported in *X. campestris* pv. vesicatoria [4], [57], [63], [65]. To identify the putative RsmA binding sites in the *hrp/hrc* transcripts, we analyzed the presence of the GGA motifs in the 5′ leader sequences of the *hrpB*, *hrpC*, *hrpD*, *hrpE*, *hrpF*, *hrpG*, and *hrpX* transcripts of XCC (Fig. 3B). We were able to find putative RsmA binding sites in the 5′ leader sequences of the *hrpC*, *hrpD*, *hrpE* and *hrpG* transcripts. No GGA motifs were found in the 5′ UTRs of *hrpB*, *hrpF* or *hrpX* (Fig. 3B).

### RsmA directly interacts with the 5′ UTRs of *hrpG* and *hrpD*

To experimentally determine whether RsmA of XCC directly binds to the GGA motifs identified in the leader sequences of *hrpC*, *hrpD*, *hrpE* and *hrpG* transcripts, RNA gel mobility shift assays were performed. RNA gel mobility shift assays were also conducted to test whether RsmA binds to the leader sequences of *hrpB*, *hrpF* and *hrpX*, which do not contain putative RsmA binding sites using RNA oligonucleotides designed based on the predicted secondary structures of the *hrpB*, *hrpF* and *hrpX* 5′ leader sequences by Mfold analysis (Table S4) [66].

**Figure 4:**
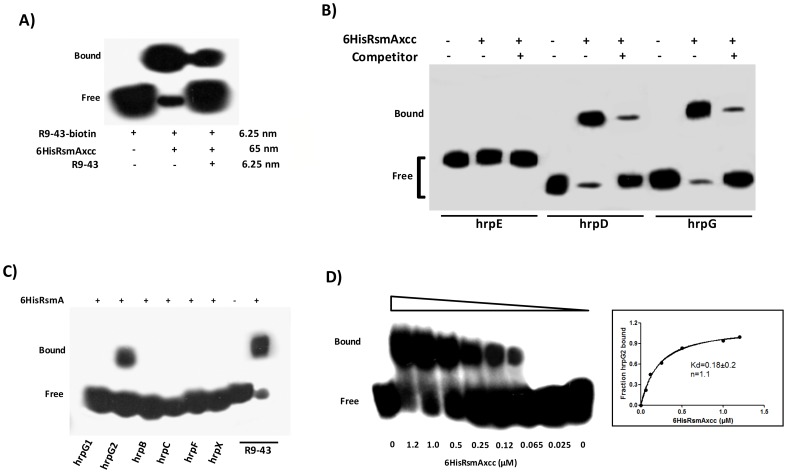
RNA mobility shift assays with purified 6HisRsmA of *X. citri* subsp. citri **A**) 6HisRsmAxcc (65 nM) binds to the high affinity RNA target R9-43. Biotin 3′-end-labeled R9-43 (6.25 nM) was incubated with 6HisRsmAxcc (65 nM) for 30 minutes at room temperature, followed by analysis on a 5% native polyacrylamide gel. A competitive assay in which unlabeled R9-43 RNA (6.25 nM) was added to the reaction reduced the signal resulting from the biotinylated nucleotide. **B**) 6HisRsmAxcc directly interacts with the 5′ UTRs of *hrpG* and *hrpD*. The leader sequences of *hrpD*, *hrpE* and *hrpG* cloned were transcribed *in vitro* and biotinylated with RNA Labeling kit (Roche). Biotinylated RNA probes were incubated with 6HisRsmAxcc and resolved in a 5% native polyacrylamide gel. The addition of unlabeled competitor R9-43 to the reactions reduced the intensity of the shifted band, which confirmed the specificity of the RsmAxcc-*hrpG* and RsmAxcc-*hrpD* interactions. **C**) 3′-end-biotin-labeled RNA probes encoding the leader sequences of *hrpB*, *hrpC*, *hrpF* and *hrpX* were tested for interactions with 6HisRsmAxcc (Table S4). In addition, 3′-end-biotin–labeled RNA probes *hrpG1* and *hrpG2*, which bear the GGA motifs encoded by the 5′ leader sequence of *hrpG*, were used to map the interaction RsmAxcc-*hrpG*. Only the GGA motif between nucleotides 80 and 120 in the *hrpG* leader sequence (*hrpG*2 probe) interacted with 6HisRsmAxcc. **D**) To determinate the apparent equilibrium binding constant (K_d_), 3′ end-labeled *hrpG*2 RNA (6.25 nM) was incubated with increasing concentrations of 6HisRsmAxcc as noted at the bottom of each lane. The binding curve for the 6HisRsmAxcc-*hrpG*2 interaction was determined as a function of 6HisRsmAxcc concentration and shifted band intensity. The average pixel value of each shifted band was calculated with ImageJ software [68], [69], [112]. The apparent equilibrium binding constant (K_d_) for this reaction was 0.18±0.2 µM 6HisRsmAxcc. Samples were loaded and resolved onto a 5% native polyacrylamide gel. All probes were transferred and cross-linked to a nylon membrane, incubated with streptavidin conjugated with horseradish peroxidase, and detected according to manufacturer's instructions (LightShiftChemiluminescent RNA EMSA Kit, Thermo Scientific). Signals + and − correspond to the presence and absence in the reaction, respectively. Positions of bound and free probes are shown.

The recombinant protein 6HisRsmA was purified by nickel affinity chromatography and tested for binding to a 3′-end-biotin-labeled R9-43 probe, which was previously identified as an *E. coli* RsmA high-affinity ligand (Table S4) [35]. Our results demonstrated that the XCC 6HisRsmA was able to promote a mobility shift of the 3′-biotin-labeled R9-43 RNA probe (Fig. 4A). We then performed the competition assay by adding unlabeled R9-43 RNA in the reaction, which led to a reduction in the intensity of the shifted band, confirming the specificity of the 6HisRsmA/R9-43 interaction (Fig. 4A).

Next, gel mobility shift analyses of the interactions between 6HisRsmA and biotinylated *hrp/hrc* leader RNA probes were performed at a concentration at least ten-fold lower than the lowest protein concentration used in the binding reactions as in Yakhnin *et al.* [67]. This enables us to assume that the concentration of free 6HisRsmA remained constant during the binding reaction [34], [35]. Interestingly, the recombinant protein 6HisRsmA caused mobility shift in the *hrpG* and *hrpD* biotinylated transcripts (Fig. 4B). 6HisRsmA did not interact with the *hrpC* or *hrpE* transcripts, which also contain potential GGA motifs in the 5′ untranslated mRNA sequences, or with *hrpB*, *hrpF* or *hrpX*, which do not contain GGA motifs in their 5′ UTRs (Figs. 4B and 4C). The biotinylated RNA probe R9-43 was used as a positive control in these assays (Fig. 4C).

Furthermore, the specificities of RsmA-*hrpG* and RsmA-*hrpD* 5′ UTRs interactions were investigated by performing competition experiments using the unlabeled specific competitor R9-43 (Fig. 4B). Also, the RsmA-*hrpG* 5′ UTR interaction was examined by gel mobility shift using two different biotinylated RNA oligonucleotides *hrpG1* and *hrpG2* (Table S4), which bear two potential RsmA binding sites found in the *hrpG* leader sequence. The recombinant protein 6HisRsmA was able to retard the *hprG*2 RNA probe (carrying the second GGA motif of the *hrpG* leader sequence), but not the *hrpG*1 RNA probe which carries the first GGA motif (Fig. 4C).

The fraction of bound *hrpG*2 increased when we raised the 6HisRsmA concentration in the binding reactions (Fig. 4D). Using the biotin-labeled *hrpG*2 probe, only one complex was observed with 0.065 µM of 6HisRsmA, and essentially most of the starting RNA was shifted with 1.2 µM of 6HisRsmA. The binding curves, Hill coefficient (n), and apparent equilibrium binding constant (K_d_) for the 6HisRsmA-*hrpG*2 RNA interaction were estimated by a nonlinear least-squares analysis using the average pixel values of the shifted bands determined with the ImageJ software [68], [69], [70] in three independent experiments, as described previously [70], [71]. The apparent K_d_ value was calculated as 0.18±0.2 µM 6HisRsmA (Fig. 4D). This value is around 3.5-fold and 7.5-fold higher than the K_d_ values of *E. coli* RsmA/CsrA when interacting with the 5′ leader regions of *cstA* (K_d_ 40 nM) and *flhDC* or *pgaA* (K_d_ ∼21 nM), respectively [44], [70], [72].

The RsmA binding site predicted within *hrpG*2 -AGAUGGAUUU- has an 80% nucleotide identity with the RsmA target site in the high-affinity ligand sequence R9-43 -ACARGGAUGU-. As the interaction of RsmA-RNA depends on RNA sequence and secondary structure and because *hrpG*1 and *hrpG*2 RNA probes bear only partial sequences of the *hrpG* leader region, our data could not rule out the possibility that RsmA also can interact with the potential binding site 1 in the *hrpG* 5′-UTR.

### Determination of the potential RsmA binding sites in the *hrpG* transcript

**Figure 5:**
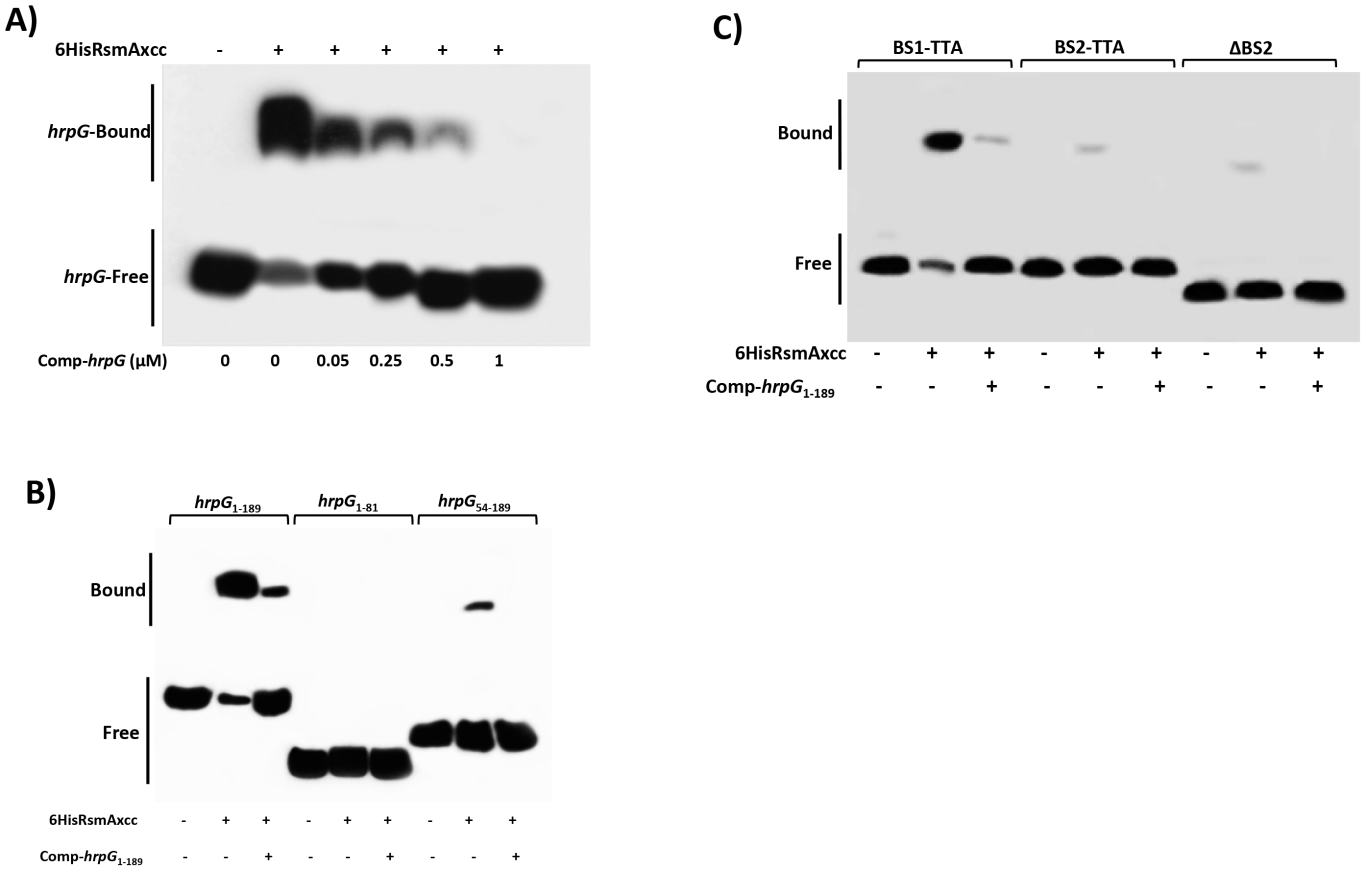
Gel mobility shift analysis for mapping the RsmA binding sites within the *hrpG* transcript. **A**) For a competition assay, interactions between 6HisRsmAxcc (0.5 µM) and biotinylated *hprG*_1-189_ transcript were tested in the presence of the competitor *hprG*_1-189_ non-biotinylated (Comp-*hrp*G). The specific concentrations of the unlabeled Comp-*hprG*_1-189_ are indicated at below of each lane. **B**) Biotinylated *hrpG*_1-189_ transcripts carrying mutations in the potential RsmA-binding sites 1 and 2 (BS1-TTA and BS2-TTA) or a deletion of the GGA motif plus 20 nucleotides in the BS2 (ΔBS2) were incubated with 6HisRsmA (0.5 µM) and competitor *hprG*_1-189_ non-biotinylated (Comp-*hprG*_1-189_). **C**) The interaction with 6HisRsmA (0.5 µM) was tested using the full length *hrpG* leader sequence (hrpG_1-189_) and two fragments *hrpG*_1-81_ and *hrpG*_54-189_ *in vitro* transcribed and biotinylated. Signals + and − correspond to the presence and absence in the reaction, respectively. Positions of bound and free *hrpG* transcripts are shown.

The specificity of RsmA interaction with full length *hrpG*_1-189_ leader sequence was investigated by performing a competition experiment with unlabeled *in vitro* transcribed 5′-UTR of the *hrpG* transcript. The concentrations of 6HisRsmA and the biotinylated *hrpG* transcript were maintained at 0.5 µM and 6.25 nM, respectively, but the concentrations of unlabeled *hrpG* transcript were ranged from 0 to 1 µM. The unlabeled *in vitro* transcribed 5′-UTR of the *hrpG* transcript was able to compete for 6HisRsmA binding as indicated by the disappearance of the mobility shift at higher concentrations of the competitor (Fig. 5A). Also, the nonspecific yeast tRNA was added to all reactions to verify the specificity of the *Rsm*A-*hrpG* transcript interaction. These results strengthen our hypothesis that RsmA binds specifically to the leader sequence of *hrpG* mRNA in XCC.

To determine whether the potential RsmA-binding sites were responsible for the observed mobility shift, we used mutated *hrpG*_1-189_ transcripts in which the GGA binding sites (BS1 and BS2) were mutagenized to TTA. Interestingly, the *hrpG* transcript mutated in the potential binding site 1 (BS1) did not prevent the mobility shift by 6HisRsmA (Fig. 5B). However, a significant reduction in the mobility shift was observed when the BS2 of the *hrpG* transcript was changed to TTA (Fig. 5B). In addition, deletion of a 23 nt fragment containing the GGA motif in the BS2 (ten nt were upstream and the other ten nt were downstream of the GGA motif) led to impairment of the mobility shift of the biotinylated *hrpG* transcript (Fig. 5B).

Furthermore, different fragments of the *hrpG* leader sequence were synthesized by *in vitro* transcription, and after biotinylation they were incubated with 6HisRsmA (0.5 µM) to verify binding activity. We did not observe mobility shift of the *hrpG*_1-81_ transcript carrying BS1 of the *hrpG*. In contrast, we observed a shift for the biotinylated *hrpG*_54-189_ transcript, although it is weaker (about 8-fold less) than that of the full length *hrpG*_1-189_ transcript (Fig. 5C). Consistently, the addition of the unlabeled *hrpG*_1-189_ transcript at high concentrations in the reactions impaired the binding of both *hrpG*_1-189_ and *hrpG*_54-189_ with 6HisRsmA (Fig. 5C). These results suggest that not only the sequence, but also the secondary structure of the *hrpG* transcript are critical for its interaction with RsmA.

### Effects of RsmA on *hrpG* and *hrpD* mRNA stability

**Figure 6:**
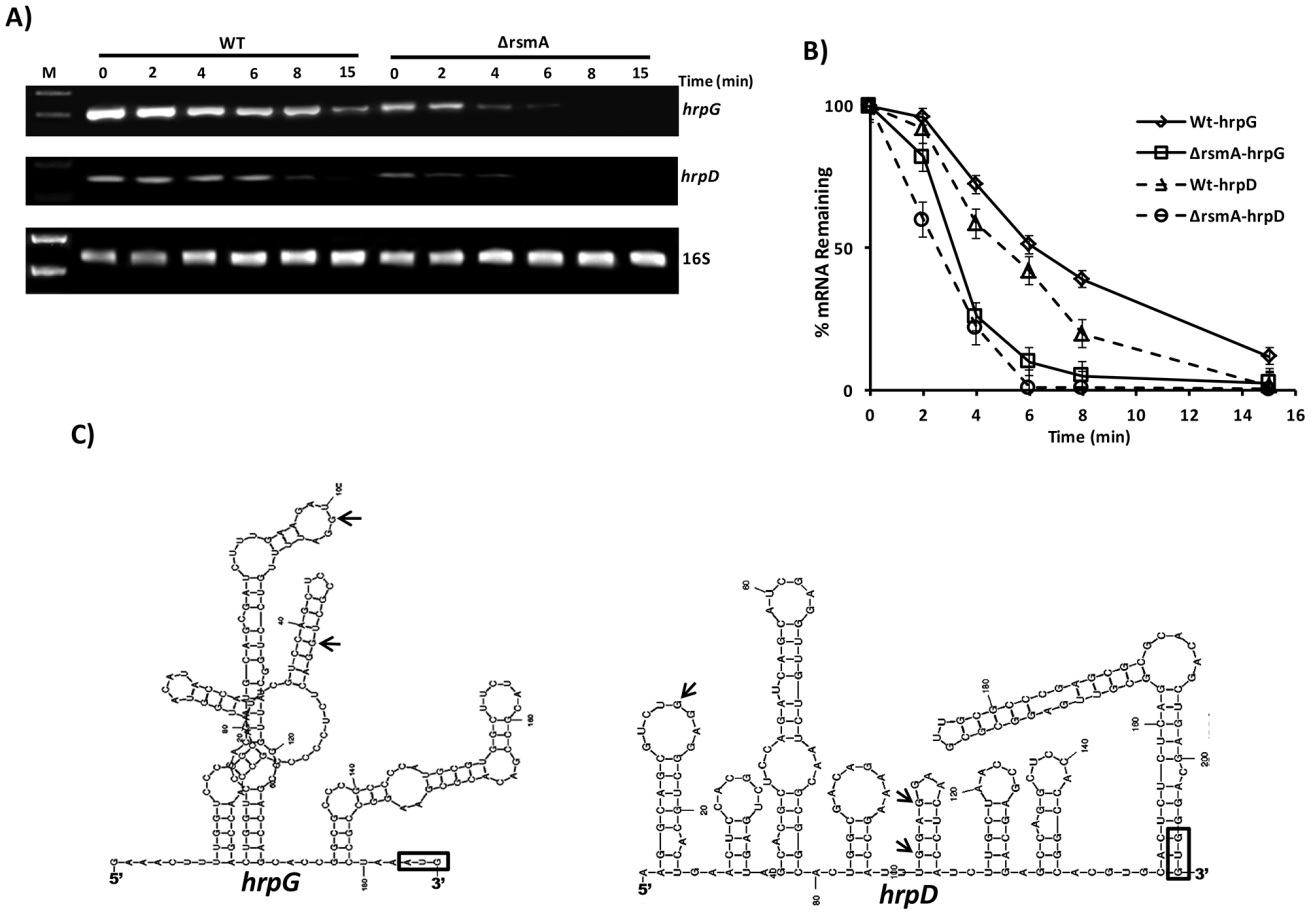
Analysis of *hrpG* and *hrpD* mRNA stability in the wild-type and *rsmA* mutant strains by RT-PCR. **A**) Cells of the XCC wild-type and Δ*rsmA* strains were grown in XVM2 medium to OD_600 nm_ = 0.6, treated with 10 µg/µL ciprofloxacin and harvested at several time points after treatment. Total RNA was isolated, and 2 µg of RNA was used for One Step RT-PCR (Qiagen) in 25 µL reactions. Reactions were subjected to PCR amplification for 26 cycles. Ten microliters of each reaction were resolved on a 1.5% agarose gel. The stability of the *hrpD* transcript was evaluated using primers annealing within the first orf *hrpQ*. The 16S RNA was analyzed as a control for normalizing the *hrpG* and *hrpD* amplification products. **B**) The relative values of *hrpG* and *hrpD* mRNA half-lives were estimated by determinating the average pixel value of each amplified product and subtracting the background using ImageJ software [68], [112]. The mean values were normalized to the corresponding 16S amplification product. Mean values derived from two independent experiments are shown. **C**) Model for the predicted secondary structure of the *hrpG* and *hrpD* leader sequences obtained with MFold software [66]. The positions of GGA motifs in the structures are indicated with arrows. AUG is shown in an open box.

As was demonstrated for *flhDC* in *E. coli*, the interaction of RsmA with the leader region of *hrpG* suggests that RsmA may stabilize and protect *hrpG* transcripts against 5′-end- dependent RNase E cleavage in XCC [44], [73], [74]. Previous studies revealed that differences in the steady-state levels of *flhDC* and *glgC* transcripts between *E. coli* wild-type and *rsmA* mutant strains are resulted from the effect of RsmA on the rates of mRNA decay [43], [44], [75]. To determine whether the stability of the *hrpG* and *hrpD* transcripts are affected by *rsmA in vivo*, the abundances of the *hrpG* and *hrpD* transcripts and the 16S transcript as a control were analyzed by RT-PCR after the addition of 10 µg/µL ciprofloxacin (Sigma, USA) in XCC cell cultures (Fig. 6A). Ciprofloxacin targets DNA gyrase and topoisomerase IV on DNA, forming ternary complexes that block the movement of replication forks and transcription complexes [76], [77]. We used ciprofloxacin in this assay because *Xanthomonas* spp. exhibit resistance to rifampicin [78], [79]. The relative abundance of the *hrpG* and *hrpD* transcripts was estimated from the specific PCR products on the agarose gel by calculating the average pixel values of the bands, subtracting the background, and determining the area of the bands by integration in the ImageJ software [68], [69], [71]. The calculated average pixel values of *hrpG* and *hrpD* were normalized based on the pixel value of the corresponding 16S amplified band, the transcript levels of which did not vary in response to the treatment. The *hrpG* and *hrpD* transcripts were at least two-fold more stable in wild-type XCC (*hrpG* and *hrpD* half-life ∼7.8 min and 6 min, respectively) than in the Δ*rsmA* mutant cells (*hrpG* and *hrpD* half-life ∼3.2 min and ∼2.8 min, respectively) (Fig. 6B), which likely contributes to the differences in the transcript levels between the Δ*rsmA* mutant and the wild-type XCC strain observed in the microarray and qRT-PCR experiments (Table 1).

The 5′ UTRs of *hrpG* and *hrpD* are 182 nt and 204 nt in length, respectively, which are considered unusually long for bacterial transcripts. RNA structure predictions using Mfold [66] suggested that the *hrpG* and *hrpD* 5′ UTRs were highly structured, containing loops (Fig. 6C). Additionally, these results suggest that *hrpG* and *hrpD* might be targeted by small RNAs in Xanthomonads. The RsmA binding site identified in the 5′ UTR of *hrpG* was found in a loop between nucleotides 80 and 120 (Fig. 6C). The predicted structure of *hrpD* 5′ UTR exhibited three GGA motifs between the nucleotides 1 and 120 and within two different loops (Fig. 6D).

### RsmA positively regulates HrpG and HrcQ protein levels

The experiments above showed that RsmA can bind and stabilize the *hrpG* and *hrpD* transcripts (Figs. 4B and 6A). The first ORF encoded by the *hrpD* operon (*hrcQRShpaA*) is *hrcQ* (Fig. S1), which encodes for an essential C-ring component of the T3SS [80]. Recent findings suggest that HrcQ might act as a general substrate acceptor for T3SS effector proteins due to its interactions with the T3S-ATPase HrcN, the T3S-membrane proteins HrcV and HrcU, and the T3SS effectors [80].

**Figure 7:**
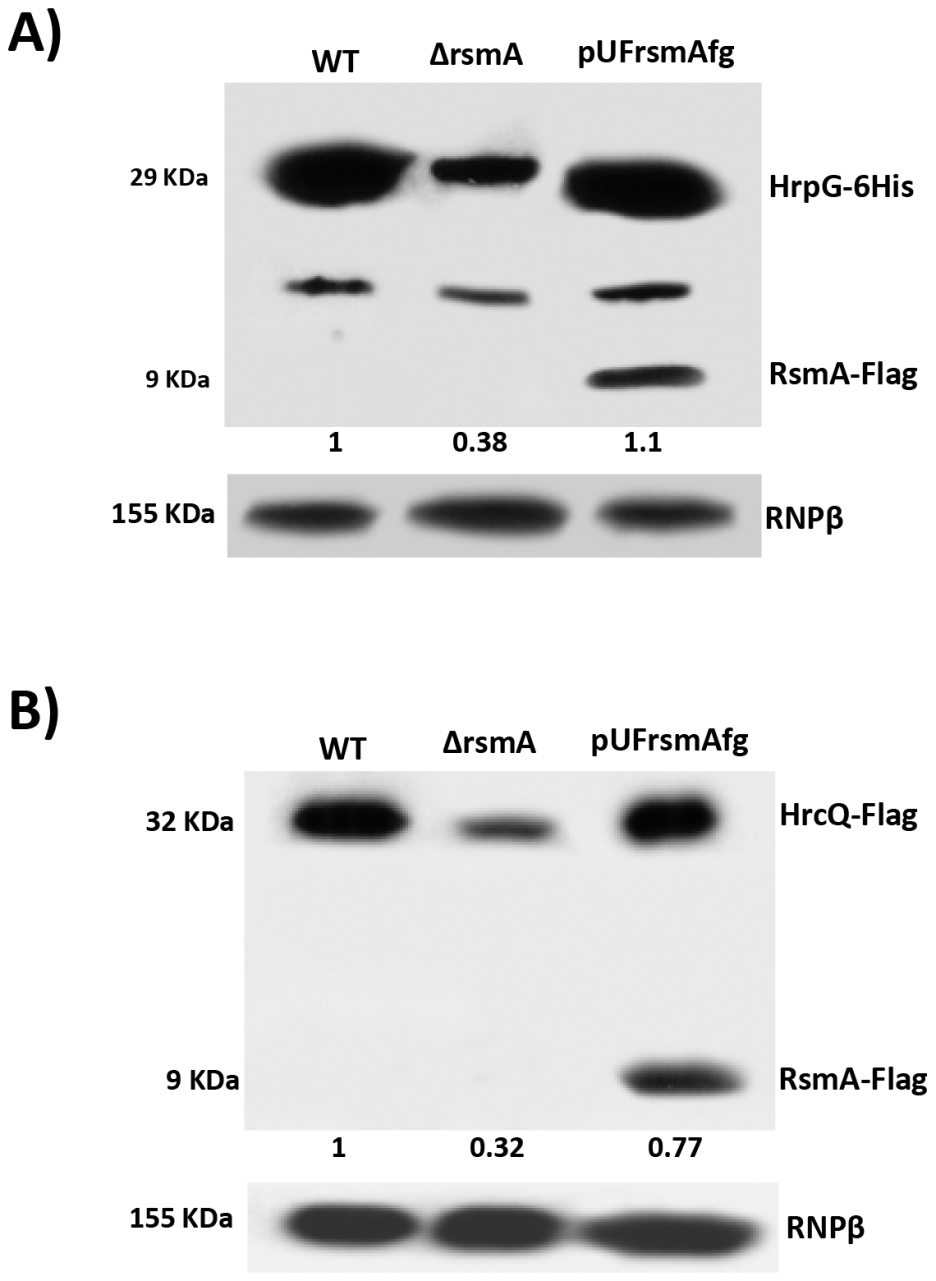
Immunoblotting experiments showed reduction of HrpG-6His and HrcQ-Flag protein levels in the *rsmA* mutant cells of *Xanthomonas citri* subsp. citri. The wild-type, Δ*rsm*A and complemented strains of XCC carrying the chromosomally inserted recombinant constructs pPM7-*hrpG*-6His and pPM7-*hrcQ*-Flag were grown in XVM2 medium to OD_600 nm_ = 0.6. Total cell extracts were analyzed by SDS-PAGE and immunoblotting using specific antibodies. **A**) For detection of HrpG-6His, after transferred to membrane protein extracts were incubated with anti-6HisTag antibodies (Medical and Biology Lab, MBL). Also, expression of RsmA-Flag was verified only in protein extracts of complemented strain (pUFrsmAfg) probed with anti-FlagTag antibodies (Sigma). **B**) For detection of HrcQ-Flag, protein extracts were incubated with anti-FlagTag antibodies (Sigma).

To verify whether the deletion of *rsmA* in XCC leads to reductions in HrpG and HrcQ protein levels, recombinant constructs encoding HrpG-6His and HrcQ-Flag with native promoters and full-length 5′ UTRs were inserted into the chromosomes of wild-type and the Δ*rsmA* mutant XCC strains using the integrative plasmid pPm7g [81]. The plasmid pPm7g harbors an 800 bp fragment of the α-amylase gene (106–912) of XCC (XAC0798), and its insertion into the XCC genome did not affect pathogenicity [81]. Additionally, Δ*rsmA* carrying the HrpG-6His construct was transformed with a plasmid-borne pUFR*rsmA*-flag to generate a complementation strain. All strains were grown in the XVM2 medium for 20 h, and total proteins were extracted for Western-blotting analysis. The deletion of *rsmA* resulted in reduced levels of HrpG-6His and HrpQ-Flag to 38% and 32%, respectively, in relation to the wild-type protein levels (Figs. 7A and 7B, respectively). The complemented strain restored the HrpG-6His and HrpQ-Flag levels to that of the wild-type strain (Fig. 7A and 7B). The relative protein values estimated for HrpG-6His and HrpQ-Flag were normalized with the bands detected in the total protein extracts probed with antibodies against the β-subunit of *E. coli* RNA polymerase (Abcam, ab12087), which was used as a loading control (Figs. 7A and 7B).

### Ectopic expression of *hrpG* in the Δ*rsmA* mutant restores its virulence and HR-triggering activity

**Figure 8:**
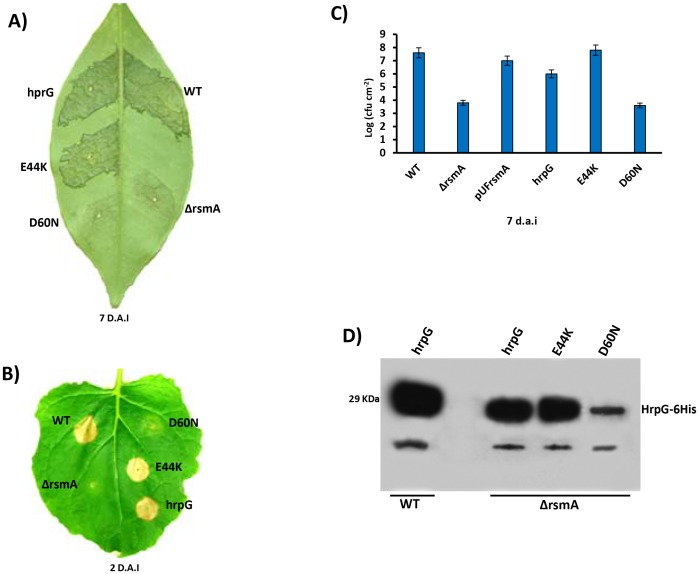
Ectopic expression of *hrpG* under control of a constitutive promoter restores full pathogenicity and HR of the *rsmA* mutant of *Xanthomonas citri* subsp. citri. **A**) XCC strains 306 (WT) and the *rsmA* mutant carrying the empty plasmid pBBR5 (ΔrsmA) or pBBR5 with *hrpG* wild-type (hrpG) or *hrpG* alleles with E44K and D60N mutations were inoculated into **A**) sweet orange (*Citrus sinensis*) leaves or **B**) tobacco leaves by infiltrating bacterial cells at a concentration of 10^6^ CFU/ml. Sweet orange leaves inoculated with WT and *rsmA* mutant strains harboring both *hrpG* and hrpG-E44K alleles showed canker symptoms 7 days after inoculation, while a strong HR was observed in tobacco leaves only 2 days after infiltration. However, constitutive expression of the hrpGD60N allele was not able to recover the pathogenicity and HR in the *rsmA* mutant. **C**) *In plant* growth curve experiments confirmed that the *rsmA* mutant transformed with both empty pBBR5 (ΔrsmA) and pBBR5Lac-*hrp*GD60N-6His (D60N) have impaired growth in host plants. Error bars represent standard deviations. **D**) Western-blotting analysis of HrpG-His protein levels in the wild-type and *rsmA* mutant strains carrying the *hrpG* (hrpG) or mutated *hrpG* alleles (E44K and D60N) under control of a constitutive promoter. Equal amounts of total cell extracts were analyzed by immunoblotting with anti-6HisTag antibodies (MBL, USA).

The results presented above suggest that RsmA contributes to the virulence of XCC by stabilizing the transcripts of *hrpG*, the master regulator of T3SS, and it also has a direct effect on the expression of *hrpD* operon. To further test our hypothesis that RsmA mostly exerts its regulation of the *hrp/hrc* genes via *hrpG*, we ectopically expressed *hrpG*-6His in the Δ*rsmA* mutant of XCC. The ectopic expression of *hrpG*-6His in the XCC Δ*rsmA* mutant not only restored its virulence and bacterial population levels in the host plant sweet orange, but also contributed to HR elicitation ability in the non-host plant *N. benthamiana* (Figs. 8A, 8B and 8C). Previously, the XCC wild-type strain 306 was shown to be able to elicit a visible HR in tobacco seven days after inoculation (Fig. 1C). However, the constitutive expression of *hrpG*-6His in the wild-type strain carrying pBBR5Lac-*hrpG*-6His can trigger a strong HR in tobacco within two days after inoculation (Fig. 8B).

To determine whether the post-translational modification of HrpG is affected by the deletion of *rsmA* in XCC, we generated mutations in the critical residues E44 and D60 within the response regulator domain of the HrpG. The *hrpG*-6His constructs bearing the E44K and D60N mutations were fused to the Lac promoter of the pBBR5 plasmid to obtain recombinant constructs pBBR5Lac-*hrpG*E44K-6His and pBBR5Lac-*hrpG*D60N-6His. It was observed that pBBR5Lac-*hrpG*-6His and pBBR5Lac-*hrpG*E44K-6His recovered the virulence and the ability to elicit HR of the Δ*rsmA* mutant (Figs. 8A, 8B and 8C). The mutant HrpGE44K was previously demonstrated to be constitutively active in *Xanthomonas* by an unknown mechanism that might involve an increase in the affinity between the HrpG response regulator domain and the α-subunit of RNA polymerase [11], [82]. On the other hand, Lac-*hrpG*D60N-6His did not restore the virulence or the HR-triggering activity of the Δ*rsmA* mutant (Figs. 8A, 8B and 8C). These findings support previous studies which had suggested that the residue D60 might be essential for phosphorylation and activation of HrpG [11]. Western-blotting experiments confirmed that HrpG-6His, HrpGE44K-6His and HrpGD60N-6His were expressed in the Δ*rsmA* mutant harboring the corresponding pBBR5 constructs, although the D60N mutant contained a lower amount of protein (Fig. 8D).

### *In vivo* phosphorylation of HrpG Asp60 residue is critical to restore the virulence of the Δ*rsmA* mutant, and the deletion of *rsmA* did not affect HrpG phosphorylation

**Figure 9:**
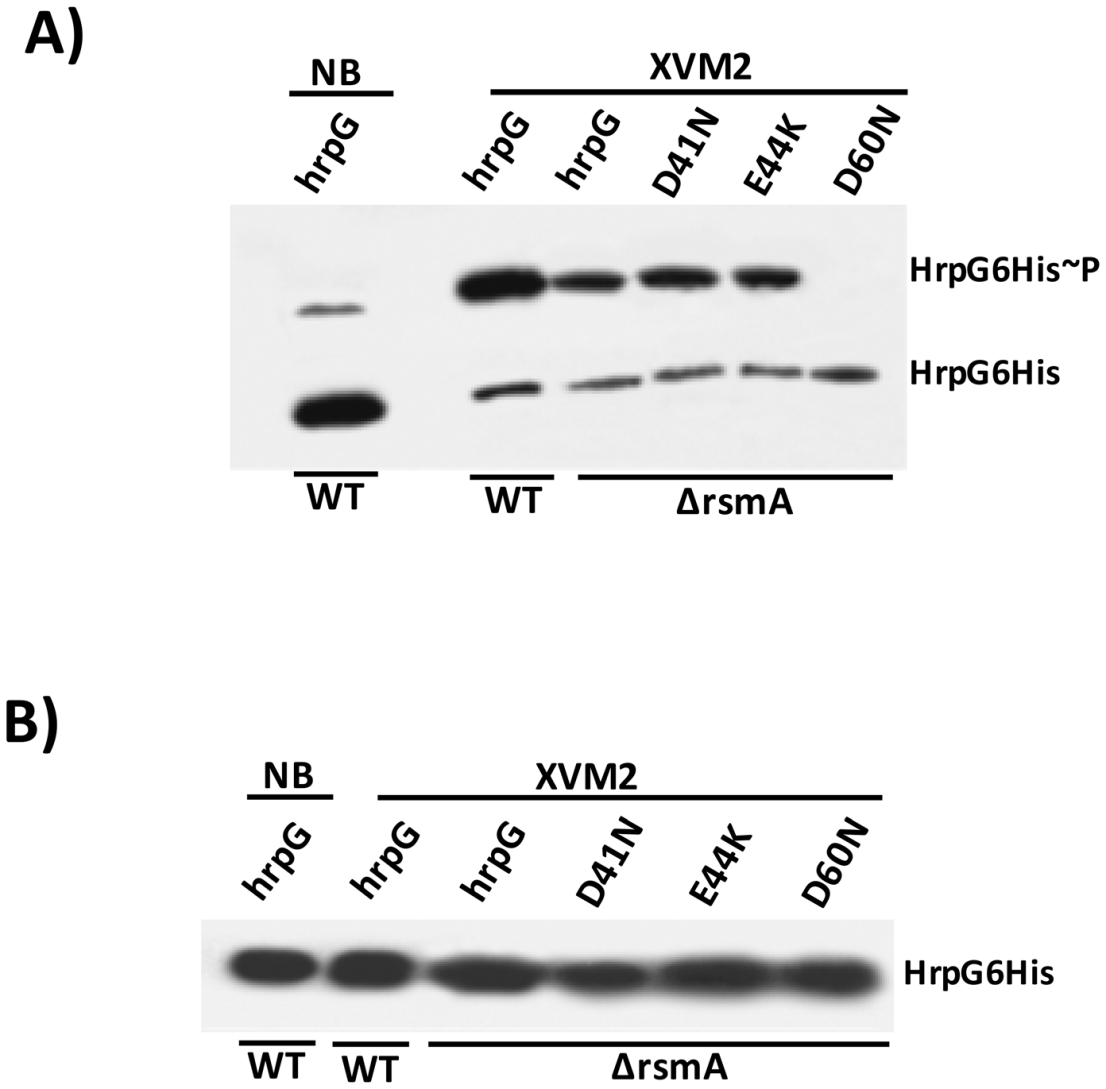
*In vivo* phosphorylation of HrpG Asp60 residue is critical to restore the virulence in the Δ*rsmA* mutant of XCC. **A**) Western-blotting analysis of XCC lysates on a 25 µM Phos-tag acrylamide gel (Wako, USA). Bacteria cells were grown in nutrient broth or XVM2 medium (T3SS inducible medium) to OD_600 nm_ = 0.6, and equal amounts of total cell extracts were analyzed by immunoblotting with anti-6HisTag antibodies (MBL, USA). **B**) Western-blotting analysis to determine HrpG-His protein levels in XCC cell extracts resolved in a 12% acrylamide gel system without Manganese(II)-Phos-tag. Strains analyzed: WT harboring the wild-type *hrpG*-6His allele; Δ*rsmA* carrying the wild-type *hrp*G-6His allele and the mutated *hrpG*-6His alleles D41N, E44K and D60N. All *hrpG*-6His constructs were cloned into the pBBR5 plasmid and placed under the control of a constitutive promoter.

As shown previously, the constitutive expression of *hrpG*-6His completely restored the virulence and HR in the Δ*rsmA* mutant, although the mutated *hrpG*D60N-6His failed to elicit canker symptoms and restore impaired bacterial growth in host plants (Figs. 8A and 8C). We next tested whether the loss in the function of *hrpG*D60N-6His was caused by impaired phosphorylation in the Δ*rsmA* mutant. We utilized the Manganese(II)-Phos-tag (Wako, USA) acrylamide gel system to analyze the *in vivo* levels of HrpG6His∼P. Both HrpG-6His and HrpG-6His∼P were detected in the cytoplasm of the XCC wild-type and Δ*rsmA* mutant strains (Fig. 9A). These two HrpG6His isoforms were also present in the mutant D41N and E44K backgrounds, but only the non-phosphorylated form was observed for the D60N mutant (Fig. 9A). A small amount of HrpG6His∼P was observed in the protein extract of wild-type cells grown in the nutrient broth medium, which represses T3SS gene expression (Fig. 9A), but the major fraction of HrpG-6His was not phosphorylated under this condition. These findings suggest that T3SS activation depends on switching the phosphorylated/non-phosphorylated ratio of HrpG isoforms in *Xanthomonas* cells. Interestingly, phosphorylated isoforms of HrpG6His were observed in the XCC wild-type and Δ*rsmA* strains transformed with the *hrpG* wild-type allele, as well as in the Δ*rsmA* strain carrying the *hrpG* mutations D41N and E44K. The *hrpG* mutation D60N impaired HrpG phosphorylation under T3SS-inducible conditions. Taken together, these results indicate that the *in vivo* phosphorylation of HrpG6His occurred at the Asp60 residue and that the deletion of *rsmA* did not affect HrpG phosphorylation under T3SS-permissive conditions (Fig. 9A). The same protein samples were separated by SDS-PAGE without Manganese(II)-Phos-tag, and then analyzed by Western-blotting to detect only the HrpG-6His isoform as a control (Fig. 9B).

## Discussion

In this study, we demonstrated that *rsmA* of XCC is critical for activating full virulence in host plants and contributing to the elicitation of HR in non-host plants by positively regulating the expression of the *hrp/hrc* genes and T3SS effector genes. Microarray and qRT-PCR analyses indicated that the *hrp/hrc* genes and T3SS effector genes are down-regulated in the *rsmA* mutant (Tables 1 and S2). Western-blotting assays and GUS assays also showed that RsmA activates the *hrp/hrc* genes at the transcriptional level (Table 1), and the *hrpG* and *hrpD* genes at the post-transcriptional level (Fig. 2). These findings are consistent with the previous transcriptomic analyses of *rsmA* mutants in *X. campestris* pv. campestris and *X. oryzae* pv. oryzae [19], [20], indicating that RsmA-mediated activation of *hrp/hrc* genes and T3SS effector genes is conserved in different species of Xanthomonads.

We further showed that RsmA binds specifically to the 5′ UTRs of the *hrpG* and *hrpD* mRNAs. Using gel shift mobility assays, we revealed that RsmA binds to the second GGA motif within a loop between nucleotide 80 and 120. Consistently, mutation or deletion of this GGA motif led to diminution of the binding of the *hrpG* transcript with RsmA (Fig. 5). Interaction of the *hrpG* and *hrpD* 5′ UTRs with RsmA seems to stabilize these transcripts by protecting them from RNA decay in XCC cells (Fig. 6). Likewise, the deletion of the *rsmA* homolog in *E. coli* reduced *flhDC* mRNA stability and flagellum gene expression, resulting in impaired swimming motility [43]. A recent report demonstrated that *E. coli* CsrA binding activates *flhDC* expression by inhibiting the 5′ end-dependent RNase E cleavage pathway [44]. Although lower abundance was observed for *hrpG* and *hrpD* mRNAs in the XCC Δ*rsmA* mutant, it remains unclear whether these transcripts are targeted by the 5′ end-dependent RNase E pathway in the absence of RsmA binding, which may lead to down-regulation of *hrp* genes expression in Xanthomonads.

Studies on the regulation of protein synthesis have shown that mRNA secondary structures present in the 5′ UTR can dramatically influence both mRNA stability and translation initiation [83], [84], [85]. Therefore, protecting the 5′ UTR of mRNA might avoid endonucleolytic cleavage, a rate-limiting step for mRNA decay [85]. Furthermore, similar to *flhDC* mRNAs that lack a strong Shine-Dalgarno sequence (GGGUGG) [44], *hrpG* and *hrpD* also contain weak Shine-Dalgarno sequences (AGGCCG and AGCUGA, respectively). Thus, the interactions between RsmA and the *hrpG* and *hrpD* leader sequences can not only promote mRNA stability, but also increase the translation efficiency of these mRNAs in XCC cells. In fact, the translational fusion of *hrpG* and *hrcQ* (the first ORF of the *hrpD* operon) to the *gusA* gene driven by a constitutive promoter resulted in reduced β-glucuronidase activity in the Δ*rsmA* mutant compared to wild-type XCC cells under T3SS-permissive conditions (Fig. 2). In addition, the proteins levels of HrpG and HrcQ were reduced in the Δ*rsmA* mutant, suggesting that RsmA may also regulate translation efficiency (Fig. 7).

The results presented here support our hypothesis that RsmA exerts its positive regulation on the *hrp/hrc* genes via *hrpG*, the upmost regulator in the regulatory hierarchy of *hrp/hrc* genes. HrpG is the master transcriptional regulator of *hrp/hrc* and T3SS effector genes by controlling the expression of the second transcriptional factor HrpX, which can recognize and directly bind to the PIP-box within the *hrp* cluster and most T3SS effector genes [9], [57]. Of all the *hrp/hrc* transcripts tested here, RsmA directly interacts with the 5′UTRs of *hrpG* and *hrpD* but not with that of *hrpB, hrpC and hrpE*, all of which carry potential GGA motifs in their leader sequences, or *hrpF* and *hrpX*, which do not carry potential GGA motifs in their 5′ UTRs. Although the significance of the direct binding of RsmA to the *hrpD* operon, which contains the T3SS structural genes *hrcQ*, *hrcR*, and *hrcS*, remains to be determined, this function was not able to explain the overall regulation of RsmA on *hrp/hrc* and T3SS effector genes. Instead, *hrpG* as the master regulator of T3SS genes, could link the overall positive regulation of T3SS genes by RsmA. This hypothesis is strengthened by the observation that the constitutive expression of *hrpG*-6His in the Δ*rsmA* mutant restored its full virulence in host plants and HR-triggering activity in the non-host plant *N. benthamiana*.

Our results further indicate that RsmA controls *hrpG* mRNA stability but not the signaling pathway involving the phosphorylation of HrpG protein. The phosphorylation of HrpG is required to activate the expression of *hrpX* and regulate downstream *hrp/hrc* and T3SS effector genes [9]. Wild-type HrpG, and the mutants HrpGE44K, and HrpGD41N could all be phosphorylated, while HrpGD60N could not be phosphorylated in the XCC cells grown in the XVM2 medium. Consequently, unlike the phosphorylated wild-type, E44K, and D41N mutants, non-phosphorylated HrpGD60N lost the ability to recover pathogenicity or HR-triggering activity of the Δ*rsmA* mutant. However, we cannot rule out the possibility that the mutation D60N might reduce HrpG protein stability, resulting in a reduction in virulence and HR-triggering activity. The deletion of *rsmA* in XCC did not affect the phosphorylation of HrpG under the conditions tested here, suggesting that RsmA can control *hrpG* expression and stability but not the signaling pathway that triggers HrpG phosphorylation.

Interestingly, in *Salmonella*, the expression of the transcriptional activator of T3SS genes, *hilA*, is directly controlled by three AraC-like regulators: HilD, HilC, and RtsA [86]. The CsrA protein binds to and prevents the translation of the *hilD* mRNA [87]. However, during the infection process, the two-component system BarA/SirA activates the expression of the *csrB* and *csrC* small RNAs, which inhibit the action of RsmA/CsrA [86], [88]. Likewise, in the phytopathogenic bacteria *E. carotovora*, RsmA targets and destabilizes the transcripts that encode plant-cell-wall degrading enzymes (PCWDEs), which are crucial virulence factors, and HrpL, an alternative sigma factor that regulates the expression of T3SS genes. The small noncoding RNA *rsmB*, which is a homolog of *E. coli* and *Salmonella csrB*, inhibits RsmA and enables the translation of the PCWDE encoding genes and *hrpL* [89], [90]. Moreover, RsmA plays an important role in the interaction between *P. aeruginosa* and human airway epithelial cells, as shown by the fact that an *rsmA* mutant failed to induce actin depolymerization and cytotoxicity in epithelial cells due to the decreased transcription of several regulators of T3SS [91].

In contrast to *Enterobacteria*, *E. carotovora*, and *Pseudomonas* spp., the signaling pathway involving RsmA in Xanthomonads is poorly understood as Xanthomonads lacks the small inhibitory RNA *rsmB*. Recently, a differential RNA sequencing (dRNA-seq) approach was used to identify eight *Xanthomonas* small RNAs whose expression levels were controlled by HrpG [18]. However, it remains to be determined whether these small RNAs target and activate *hrp* genes. Furthermore, Schmidtke et al. identified transcripts with unusually long 5′-UTRs, which could indicate extensive post-transcriptional regulation in Xanthomonads [18]. The RsmA target mRNAs *hrpG* and *hrpD* identified here also contain long leader regions with loops, suggesting that the expression of T3SS genes may be under several post-transcriptional controls in Xanthomonads. Recent findings demonstrated that in *E. coli*, the 5′ UTR of the flagellum master regulator *flhDC* mRNA is targeted for repression by four small RNAs (ArcZ, OmrA, OmrB and OxyS), and for activation by one small RNA (McaS) [92], [93].

RsmA seems to positively regulate critical genes involved in pathogen-host interactions such as *hrp/hrc* and T3SS effector genes in plant pathogenic bacteria. RsmA also regulates features that are controlled by the quorum-sensing signaling pathway such as extracellular enzymes production, cell motility and biofilm formation in bacteria. Fine-tuning of RsmA activity allows bacteria to overcome a variety of environmental challenges such as host defenses by promptly modifying the mRNA stability and regulating the translation initiation of specific transcripts. Future studies designed to identify new components of RsmA regulatory circuits in Xanthomonads, such as small RNAs, two-component system proteins, and 5′ end-dependent RNAse E cleavage pathways, will shed light on the signaling network of the HrpG-HrpX-mediated expression of the T3S machinery, and also the overlapping of regulation of the virulence traits driven by the quorum-sensing system in plant pathogenic bacteria.

## Materials and Methods

### Bacterial strains, plasmids, and growth conditions

The plasmids, oligonucleotides and bacterial strains used in this study are listed in Tables S1 and S4. *E. coli* cells were grown at 37°C in lysogeny broth (LB) medium (1% (w/v) tryptone, 0.5% (w/v) yeast extract, 1% (w/v) sodium chloride, pH 7.5). The XCC wild-type strain 306 (rifampicin resistant) [46] and the mutant strains were routinely grown in nutrient broth (NB), on nutrient agar (NA) at 28°C. For induction of *hrp* genes, XCC cells were grown in the minimal medium XVM2 (pH 6.7, 20 mM NaCl, 10 mM (NH_4_)_2_SO_4_, 5 mM MgSO_4_, 1 mM CaCl_2_, 0.16 mM KH_2_PO_4_, 0.32 mM K_2_HPO_4_, 0.01 mM FeSO_4_, 10 mM fructose, 10 mM sucrose, 0.03% casamino acid) as previously described [57]. Endoglucanase assays were performed as previously described in [94], [95], but with the addition of 0.25% L-arabinose. Plasmids were introduced into *E. coli* by heat-shock at 42°C and into XCC by electroporation [96]. XCC deletion mutants were generated using the suicide vectors pNPTS138 as described elsewhere [97]. Antibiotics were used at the following concentrations: rifampicin, 50 mg/ml; kanamycin, 50 mg/mL; ampicillin, 100 mg/mL; spectinomycin, 50 mg/mL; streptomycin, 50 mg/mL; gentamicin, 10 mg/mL; chloramphenicol, 35 mg/mL; tetracycline, 10 mg/mL and ciprofloxacin 10 mg/mL.

### Construction of the *rsmA* deletion mutant and complemented strains of *X. citri* subsp. citri

DNA manipulations and PCR were performed according to standard procedures [98]. Restriction digestion and DNA ligation were performed in accordance with the manufacturer's instructions (New England Biolabs, USA). To construct the *rsmA* deletion mutant, approximately 1 kb of the upstream and downstream regions of the *rsmA* gene (*XAC1743*) were amplified by PCR from XCC genomic DNA, and the two fragments were ligated to produce an in frame deletion, leaving only the region coding for the first five and last four codons. This sequence was then cloned into the *EcoRI* restriction site of the pNPTS138 suicide vector (M. R. Alley, unpublished), thus generating the plasmid pNPTS-rsmA. This vector was introduced into XCC by electroporation, and the wild-type copy was replaced by the deleted version after two recombination events as described previously [97]. To complement the *rsmA* knockout mutant, a fragment including the *rsmA* gene plus 1000 bp of upstream sequence was amplified by PCR from XCC genomic DNA and inserted into the pUFR047 vector [99] at the *EcoRI* restriction site, creating the plasmid pUFR-rsmA. This plasmid was then transferred to the Δ*rsmA* strain by electroporation and selection for gentamicin resistance. To generate the complementation plasmid for the *hrpG* mutant, the fragment containing the entire *hrpG* gene and its promoter was amplified using XCC genomic DNA as a template and the primers F-hrpG and R-hrpG. The fragment was purified from a 1% agarose gel, digested with *Bam*HI and *Hin*dIII restriction enzymes and cloned into the *Bam*HI/*Hin*dIII enzyme sites of pUFR047. The derivative construct, pUFR-*hrpG*, was transferred into the *hrpG* mutant by electroporation (Table S1). The transformed XCC cells were selected on NA with gentamicin. All constructs were sequenced for confirmation.

### Generation of expression plasmids in XCC

To produce the fusion of *rsmA* to an L-arabinose inducible promoter (BAD) in the plasmid pBRA-6HisrsmA, pET-6HisrsmA (Table S1) was digested with the *Nco*I/*Xho*I restriction enzymes, and the DNA fragment encoding 6HisRsmA was inserted into the *Nco*I/*Sal*I enzyme sites of pBRA (Table S1). To generate a Flag epitope-tagged RsmA containing its native promoter, a fragment encoding the rsmA gene plus 1 kb upstream sequence was amplified by PCR using the primers F1-rsmAflag and R1-rsmAflag (Table S3), and then a second PCR using F1-rsmAflag and R2-rsmAflag created the fragment rsmA-flag, which was digested with the *Bam*HI and *Xho*I restriction enzymes and cloned into the *Bam*HI/*Sal*I enzymatic sites of pUFR047. The resulting construct pUFR-rsmAflag was used to transform the *XCC* Δ*rsmA* mutant strain by electroporation. To fuse *hrpG* to a constitutive promoter, the construct pBBRLac-hrpG6his was produced by PCR amplification of the *hrpG* orf plus 50 nt upstream using the Fwconst-hrpG and R2-hrpG6His primers (Table S3). The generated fragment was digested with the *Kpn*I/*Xho*I restriction enzymes and cloned into the same enzymatic sites of pBBR1-MCS-5. The plasmid pBBR1-MCS-5 contains a *lac* promoter that promotes constitutive gene expression in *Xanthomonas* [100]. The construct pBBRLac-hrpG6his was then used as a template to produce the HrpG point mutants *hrcGD41N-6His*, *hrcGE44K-6His and hrcGD60N-6His* using the QuikChange Site-Directed Mutagenesis Kit and the specific primers as described in Table S3 (Stratagene). The constructs *hrpG-6his and hrcQ-flag* with their native promoters were produced by two parallel PCR reactions using XCC genomic DNA as the template and the primers F-hrpG/R1-hrpGflag and F-hrcQ/R1-hrcQflag, respectively. In a second PCR, the *hrpG* and *hrcQ* fragments amplified in the previous step were used as templates with the primers F-hrpG/R2/hrpGflag and F-hrcQ/R2-hrcQflag generating the restriction enzyme sites within the amplified fragments (Table S3). The recombinant constructs *hrpG-his and hrcQ-Flag* were digested and cloned into the *Bam*HI/*Xho*I enzymatic sites of the pPm7g plasmid producing hrpG6his-pPm7g and hrcQflag-pP7mg (Table S1). These pP7mg constructs were used to insert hrpG6his and hrcQflag into the XCC chromosome. The pP7mg plasmid harbors an 800 bp fragment extracted from the α-amylase gene (106–912) of XCC (XAC0798), and its insertion into XCC chromosome did not affect pathogenicity [81]. XCC cells transformed with pPM7-*hrpG*-6His and pPM7-*hrcQ*-Flag constructs were selected in NA with kanamycin and confirmed by PCR. All constructs were confirmed by sequencing.

### Pathogenicity and HR assays

Pathogenicity assays were conducted in a quarantine greenhouse facility at the Citrus Research and Education Center, Lake Alfred, FL, U.S.A. Assays were performed using fully expanded, immature leaves of sweet orange (*Citrus sinensis*). XCC wild-type and mutant strains used in this assay were grown with shaking overnight at 28°C in NB, centrifuged, and suspended in sterile tap water, and the concentrations were adjusted to 10^8^ CFU/ml. For the pathogenicity assays, bacterial solutions of both 10^8^ and 10^5^ CFU/ml were infiltrated into the leaves with needleless syringes [9], [101]. Disease symptoms were photographed at 7 days post-inoculation. The strains were also tested for their ability to elicit an HR on *Nicotiana benthamiana* by infiltration of plant tissue with strains adjusted to 10^8^ CFU/ml with a needleless syringe. Plant responses were scored for HR in tobacco 2 days and 7 days post-inoculation. Tobacco plants were grown in growth chambers at 25°C with a 12 h photoperiod. Experiments were repeated three times with similar results.

### RNA extraction and qRT-PCR assays

Single bacterial colonies were picked and grown in 5 ml of NB at 28°C for 24 h with shaking, and then transferred into 50 ml of NB for overnight incubation. The bacterial cultures in the middle exponential stage were centrifuged, washed twice with XVM2 medium, and then inoculated in XVM2 medium at an initial concentration of OD_600 nm_ 0.03. Bacteria were grown in XVM2 medium or XMV2 plus 0.25% L-arabinose with shaking at 200 rpm at 28°C, and samples of the cultures were collected at OD_600 nm_ 0.5. Four biological replicates were used for each strain for qRT-PCR and microarray analyses. RNA was stabilized immediately by mixing the bacterial culture with two volumes of RNA protect bacterial reagent (Qiagen, CA, U.S.A.) and incubated at room temperature for 5 min. Bacterial cells were centrifuged at 5000× *g* for 10 min and cell pellets were used for RNA extraction. Cell pellets were treated with lysozyme, and RNA extractions were performed using an RNeasy Mini kit (Qiagen). Purified RNA was treated with DNAse I-Free (Qiagen), and successful removal of genomic DNA was confirmed by PCR. RNA quantity was initially determined on a ND-8000 Nanodrop spectrophotometer (NanoDrop Technologies, U.S.A.) and RNA quality was assessed using the Agilent 2100 bioanalyzer (Agilent Technologies, CA, U.S.A.). For qRT-PCR assays, reverse transcription was performed using 1 µg of DNaseI-treated RNA and the RevertAid H-Minus First Strand cDNA Synthesis Kit, following the manufacturer's protocol (Fermentas). Quantitative amplification of the resulting cDNA (40 ng) was performed using 0.3 µM of each primer (Table S4) and SYBR Green/ROX qPCR Master Mix in the ABI7300 Real-Time System (Applied Biosystems). Relative quantification of gene expression was performed using 16S and *XAC1631* (which codes for subunit A of DNA gyrase) as endogenous controls and the 2^−ΔΔCT^ method [102]. Primers were designed using the Primer Express Software (Applied Biosystems).

### Microarray analyses

For transcriptome studies of the Δ*rsmA* mutant and Δ*rsmA* mutant carrying pBRA-*rsmA*, an Agilent 8-by-15K microarray containing 60-mer oligonucleotides for all predicted orfs in the XCC strain 306 genome were used as described previously [9]. Labeled cDNA was generated using a Fairplay III microarray labeling kit (Agilent Technologies). Total RNA input (5 µg) was used to generate labeled cDNA according to the manufacturer's protocol. Briefly, cDNA was synthesized from 5 µg of the total RNA with Affinity Script HC and random primer, and then modified cDNA was labeled with either cy3 or cy5. The labeled cDNA was purified following the manufacturer's instructions [9], [103]. A total of 300 ng of labeled cDNA per sample was used for the hybridization as previously described [9]. A dye swap was performed to remove any bias resulting from the labeling dyes. Hybridization was performed using a Gene Expression Hybridization Kit (Agilent Technologies) according to microarray protocols recommended by Agilent. The arrays were scanned using a dual-laser DNA microarray scanner (Model G2505C; Agilent Technologies). The microarray analyses, normalization of the mean signal intensities and statistics of the data were carried out as described previously [9]. Log2-transformed values were used for statistical analysis. A linear modeling approach and the empirical Bayes statistics for differential expression analysis were employed as in the report by [104]. The *P* values were adjusted using the Benjamini and Hochberg method, designated as FDR [105]. Differentially expressed genes were ranked based on FDR, and genes with FDR<0.05 and a minimum absolute value of log2-fold-change > = 1 (equivalent to 2-fold) were considered to be significantly differentially expressed.

### Western-blotting and *in vivo* phosphorylation analyses

To analyze the protein levels in XCC, cells were grown in XVM2 medium to an OD_600 nm_ of 0.5. Bacterial cells were harvested and pellets were resuspended in 50 mM Tris-HCl (pH 8.0), 100 mM NaCl, 0.1% Tween-20, and 1 mg ml^−1^ lysozyme. Cells were ruptured by sonication. Cell-free extract was prepared by centrifugation at 15, 000 *g* at 4°C for 45 min. Protein extracts were quantified by the BCA protein assay (Pierce). Equal amount of proteins were loaded and separated by 15% SDS-PAGE, transferred to a nitrocellulose membrane (GE Healthcare) followed by immunoblotting analysis. Rabbit polyclonal antibodies raised against XCC HrcU, HrpB1, HrpB2 and HrpD6 were previously reported in Cappelletti *et al.* [57]. The rabbit sera against Hrp proteins were diluted to 1∶1000, while anti-Flag (Sigma) and anti-6His (BML, USA) were diluted to 1∶5000. Rabbit antibodies were detected with staphylococcal protein-A conjugated to horseradish peroxidase (Sigma). The *in vivo* levels of phosphorylated HrpG were detected using the Manganese(II)-Phos-tag (Wako, USA) acrylamide gel system. Formic acid-lysed XCC cells grown in XVM2 media to mid-log phase (OD_600 nm_ = 0.6) at 30°C were fractionated on a 12.5% acrylamide gel as described previously [106], [107]. After electrophoresis, the gel was washed twice with transfer buffer plus 5 mM EDTA pH 8, and the resolved proteins were then transferred to a nitrocellulose membrane. The membrane was then incubated with anti-6HisTag antibody (BML, USA) followed by protein A-peroxidase. As a control to these experiments, immunoblotting was carried out for the same protein extracts resolved on a 12.5% acrylamide gel without Manganese(II)-Phos-tag. Immunoblots were developed with SuperSignal West Femto Chemiluminescent Substrate (Pierce) and exposed to CL-XPosure Film (Pierce).

### GUS activity assay

To generate the translational constructs with *hrp* gene-*gusA* fusion under control of the *hrp* genes native promoters, the promoter and 5′ UTRs plus three first codons of the *hrp* genes were fused in frame to the promoterless *gusA* gene without its ribosome binding site [108]. The upstream sequence and first codons of the *hrp* genes were amplified by PCR using the total DNA of the XCC wild-type strain 306 as the template. The *gusA* orf was amplified from the vector pBI121 (Table S1). The primer pairs used in these PCR reactions are listed in Table S3. Fragments containing the translational *hrp* gene-*gusA* fusions were first cloned into the pGEM T-easy vector, and then digested with *Eco*RI/*Hin*dIII and inserted into the same enzymatic sites of pLAFR3 [109] (Table S1). Further, the translational constructs with *hrp* gene-*gusA* fusion driven by a constitutive promoter were produced after amplifying the leader sequence of *hrp* genes plus *gus*A from the constructs described above (Table S3). The PCR products were purified, digested with *Bam*HI/*Hind*III and inserted downstream of the *lac* promoter in the vector pUC18-mini-Tn7T-LAC [110]. Those constructs were used to transform XCC wild-type and *rsmA* mutant strains. Transformed colonies were selected on NA medium plus tetracycline or gentamicin. Wild-type and *rsmA* mutant cells harboring plasmid-borne promoterless *gusA* fused in frame to the *hrp* genes were cultured in XVM2 for 20 h and assayed for β-glucuronidase (GUS) activity. Bacteria cells were diluted and disrupted in a GUS assay buffer (20 mM Tris-HCl, pH 7.0, 10 mM 2-mercaptoethanol, 5 mM EDTA, and 1% TritonX-100). GUS activities were determined at intervals of 15 min for 4 h by measuring the OD_420 nm_ using PNPG (p-Nitrophenyl-β-D-glucuronide) as the substrate [60]. The relative GUS activity was expressed as (1,000×A420)/(time in min×OD600) in Miller units (U) [27], [111]. Values presented are means ± standard deviations of three independent experiments. The GUS assay was repeated twice with similar results.

### 5′-RACE

The transcriptional start sites of *hrpG* and *hrpX* were determined using the 3′/5′ RACE Kit (Roche), according to manufacturer's instructions. Briefly, total RNA was obtained from XCC wild-type cell cultures grown in XVM2 medium to an OD_600 nm_ of 0.5. After treatment with DNase I-free (Qiagen), the RNA was reverse transcribed using a gene-specific primer (SP1, Table S4), purified and poly(dA) tailed at the 3′ end by reaction with terminal transferase enzyme. The resulting cDNA was amplified by PCR using the poly-dT primer provided by the kit to anneal at the poly(dA) tail and a gene-specific primer (SP2, Table S3), complementary to a region upstream of the original cDNA primer. The amplicons from the first PCR were submitted to a second PCR reaction using the poly dT primer and a distinct gene-specific nested primer (SP3, Table S3) that was internal to the first. PCR products were ligated into the pGEM-T vector (Promega) and several distinct clones were sequenced.

### Expression and purification of 6HisRsmA in *E. coli*

For expression of the recombinant protein 6HisRsmA in *E. coli*, a fragment containing the *rsmA* gene sequence was amplified by PCR from XCC genome using primers and F-peTrsmA and R-peTrsmA (Table S4), digested with *Nde*I/*Eco*RI and inserted into pET-28a (Novagen) resulting in pET-6HisrsmA. Recombinant 6HisRsmA protein was over-expressed in *E. coli* BL21 Star (DE3) pLysS (Novagen) by addition of 1 mM isopropyl-b-D-thiogalactopyranoside to cells grown to mid-exponential phase. Bacterial cells were harvested 4 h later. Cell pellets were resuspended in 50 mM NaH2PO4 (pH 8.0), 300 mM NaCl, 10 mM imidazole and 1 mg ml^−1^ lysozyme. Cells were ruptured by sonication. Cell-free extract was prepared by centrifugation at 14,000 *g* at 4°C for 45 min. 6HisRsmA was purified by Ni-NTA affinity chromatography (Pierce) according to the manufacturer's recommendations. The protein-containing fractions were concentrated and the buffer was exchanged with 10 mM HEPES pH 7.3, 20 mM KCl, 1 mM MgCl2, 1 mM DTT, and 10% Glycerol. The protein concentration was estimated using a BCA method (Pierce) with bovine serum albumin as the standard. The purified protein was stored at −20°C.

### RNA gel mobility shift assay

RNA gel mobility shift assays were carried out as previously reported by Yakhnin *et al.* [67] with some modifications. The leader sequences of the *hrpD*, *hrpE* and *hrpG* were transcribed *in vitro* with the T7 RNA polymerase (Roche) and biotinylated using the Biotin RNA Labeling Mix (Roche). Further, 3′-end-biotin-labeled RNA oligonucleotides encoding the leader sequences of *hrpC*, which contain a GGA motif, and *hrpB*, *hrpF* and *hrpX*, which do not contain putative RsmA binding sites, were synthesized by IDT, Inc. (Integrated DNA Technology, Coralville, Iowa, USA) to test also for interactions with 6HisRsmA (Table S4). The RNA oligonucleotides were designed based on the predicted secondary structures of the *hrpB, hrpC, hrpF and hrpX* 5′ leader sequences by analysis in MFold [66]. Briefly, DNA templates for the generation of the targeted RNA transcripts were produced using XCC wild-type strain 306 genomic DNA as template and the specific primer pairs shown in Table S3. These DNA fragments were cloned into pGEM T-vector producing pGEMT-5UTRhrpG, pGEMT-5UTRhrpD and pGEMT-5UTRhrpE (Table S1). These plasmids were linearized with *Pst*I restriction enzyme and purified using DNA gel extraction kit (Qiagen). The *hrp* transcripts were produced *in vitro* by using the T7 Transcription kit (Roche) and Biotin RNA labeling Kit (Roche) following the manufacturer's instructions. Transcripts were purified by using the Zymoclean Gel RNA Recovery kit (Zymo Research, USA), re-suspended in DEPC water and quantified using Nanodrop. Biotin-labeled RNA probes, *in vitro* transcribed (Table S3) or synthesized by IDT corporation (Table S4), were heated to 95°C for 3 minutes and reconstituted by incubating 15 minutes at room temperature. The binding assays were performed using the LightShift Chemiluminescent RNA EMSA Kit (Thermo Scientific, USA) according to the manufacturer's instructions. Then approximately 65 nM 6HisRsmA protein and 6.25 nM Biotin-labeled RNA were mixed into a tube with binding buffer [(10 mM HEPES pH 7.3, 20 mM KCl, 1 mM MgCl2, 1 mM DTT, 5% Glycerol, 0.1 µg/µL yeast tRNA, 20 U RNasin (Promega)] to a total volume of 20 µL reaction. The binding reactions were incubated at 25°C for 20 min to allow RsmA–RNA complex formation. A total of 5 µL loading buffer (97% glycerol, 0.01% bromophenol blue, 0.01% xylene cyanol) was added to the binding reaction and immediately loaded and separated using 5% native polyacrylamide gels. Signal bands were visualized using the LightShift Chemiluminescent RNA EMSA Kit (Thermo Scientific) according to the manufacturer's instructions. In the competition assays and for calculating apparent K_d_ of RsmA-hrpG2 complex the concentrations of the 6HisRsmA and RNA probes are indicated in the respective figures and legends. The binding curve for 6HisRsmAxcc-hrpG2 interaction was defined as a function of 6HisRsmA concentrations and relative values of shifted band intensity. The apparent equilibrium binding constant (K_d_) was estimated based on the average pixel value of each shifted band calculated with ImageJ software [68], [69], [112]. The calculated mean pixel values (*P*-value <0.05) were obtained as results of three independent assays.

### mRNA stability assay

Bacterial cultures of XCC wild-type and *rsmA* mutant were grown at 28°C in XVM2 medium to OD_600 nm_ of 0.6 and treated with ciprofloxacin at a final concentration of 10 mg/ml to inhibit transcription. Samples were collected at 0, 2, 4, 6, 8 and 15 min after ciprofloxacin treatment. The cells were harvested by centrifugation at 10,000 r.p.m. and used immediately for RNA extraction by addition of Trizol (Life, USA) in the pellets, and heating at 65°C for 10 min. After the separation of organic and aqueous phase, the supernatants containing total RNA were transferred to new eppendorf tubes and RNA extraction was continued by using RNeasy Mini kit (Qiagen). Total RNA samples were treated with DNase I-Free (Qiagen) and quantified by using Nanodrop. A total of 2 µg of treated RNA was used for One-Step RT-PCR (Qiagen) in 25 µL reactions following the manufacturer's protocol. Reactions were subjected to PCR amplification for 26 cycles. Ten microliters of each reaction was resolved in 1.5% agarose gel. Stability of *hrpD* transcript was evaluated with primers annealing within the first orf *hrpQ* (Table S4). Analysis of 16S RNA was performed as control for normalizing the *hrpG* and *hrpD* amplified products. The relative value of *hrpG* and *hrpD* mRNA half-life was estimated with determination of the average pixel value of each amplified products and subtracting the background by ImageJ software [68], [112]. The obtained mean values were normalized with 16S correspondent product of amplification. Mean values derived from two independent experiments are shown.

## Supporting Information

Figure S1 **Genetic organization of** ***hrp*** **gene clusters of** ***X. citri*** **subsp.** **citri** **.** The open reading frames which form the transcript units in *hrp* cluster, *hrpA* to *hrpF*, are represented by thick arrows in the map. The PIP boxes positions in each transcript unit and their orientations are indicate by thin arrows, except to *hrpA* which does not contain a PIP box, orientation of the promoter is shown with dashed arrows. *hrc* genes encode proteins conserved among type 3 secretion systems; *hrp* and *hpa* genes encode non-conserved proteins involved with hypersensitive response and pathogenicity.
(PDF)

Figure S2 **Graphical representation of the multiple sequence alignment and structural model of RsmA of** ***Xanthomonas citri*** **subsp. citri.** **A**) Sequences of RsmA homologous proteins were used to generate this representation using the WebLogo server (http://weblogo.berkeley.edu/) [113], in which the height of the residue symbol indicates the degree of conservation and the numbers refer to residue positions in RsmA. RsmA secondary structure was predicted by Phyre (http://www.sbg.bio.ic.ac.uk/~phyre/) [50]. In of the predicted RsmA secondary structure, boxes indicate alpha-helices and arrows indicate β-strand motifs. **B**) The RsmA structure model was obtained using the Swiss-Model Repository program with the *E. coli* CsrA structure as a model [PDB: 1Y00, [48]]. The structure was generated using PyMOL [114]. The critical residues involved in RNA binding are highlighted in A) with stars and in B) with sticks in the RsmA three-dimensional structure model [33], [34].
(PDF)

Figure S3 **Induced expression of 6HisRsmA restores the production levels of endoglucanases in** ***rsmA*** **mutant of** ***X. citri*** **subsp. citri.** **A**) Expression of 6HisrsmA upon induction with 0.25% L-arabinose was confirmed by Western-blot analysis with anti-6His antiserum. *X. citri* subsp. citri strains were growth in XVM2 medium at 28°C and OD_600 nm_ 0.5, and after 4 hours of induction with 0.25% L-arabinose the cells were collect and centrifuged. Supernatants were discarded and cells were resuspended in Laemmli sample buffer. Proteins extracts were analyzed by SDS-PAGE, transferred to nitrocellulose membrane and incubated with anti-6His antiserum. *E. coli* cell extract expressing the recombinant protein 6HisRsmA was used as a positive control. **B**) Induced expression of 6HisRsmA restores the production levels of endoglucanases in *rsmA* mutant of *X. citri* subsp. citri. Endoglucanases activity assays were performed with supernatant of the strains growth in NB medium supplemented with 0.25% L-arabinose at 28°C and OD_600 nm_ 1.0. Wt, XCC strain 306; pBRA, mutant *rsmA* harboring empty plasmid pBRA; pBRA6HisrsmA, *rsm*A mutant transformed with plasmid-borne 6HisrsmA under control of an arabinose induced promoter.
(PDF)

Table S1 **Bacterial strains and plasmids used in this study.**
(PDF)

Table S2 **Comparison of expression of the genes regulated by** ***rsmA*** **in** ***Xanthomonas citri*** **subsp. citri. using microarray.**
(PDF)

Table S3 **Oligonucleotides primers used in this study.**
(PDF)

Table S4 **RNA probes encoding 5′-UTR of hrp genes and control used in this study.**
(PDF)
