# Supplementary material for: The Post-transcriptional Regulator rsmA/csrA Activates T3SS by Stabilizing the 5′ UTR of hrpG, the Master Regulator of hrp/hrc Genes, in Xanthomonas
Source: PLoS Pathog. 2014 Feb 27;10(2):e1003945. doi: 10.1371/journal.ppat.1003945 (PMC3937308; doi:10.1371/journal.ppat.1003945)
Supplement: Figure S1 — Genetic organization of hrp gene clusters of X. citri subsp. citri . The open reading frames which form the transcript units in hrp cluster, hrpA to hrpF, are represented by thick arrows in the map. The PIP boxes positions in each transcript unit and their orientations are indicate by thin arrows, except to hrpA which does not contain a PIP box, orientation of the promoter is shown with dashed arrows. hrc genes encode proteins conserved among type 3 secretion systems; hrp and hpa genes encode non-conserved proteins involved with hypersensitive response and pathogenicity. (PDF) [file ppat.1003945.s001.pdf]

Figure S1.

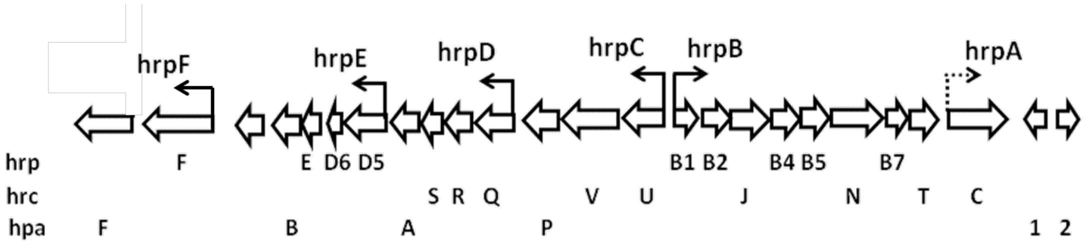

Figure S1. Genetic organization of *hrp* gene clusters of *X. citri* subsp. *citri*. The open reading frames which form the transcript units in *hrp* cluster, *hrpA* to *hrpF*, are represented by thick arrows in the map. The PIP boxes positions in each transcript unit and their orientations are indicate by thin arrows, except to *hrpA* which does not contain a PIP box, orientation of the promoter is shown with dashed arrows. *hrc* genes encode proteins conserved among type 3 secretion systems; *hrp* and *hpa* genes encode non-conserved proteins involved with hypersensitive response and pathogenicity.
