# Supplementary material for: The Post-transcriptional Regulator rsmA/csrA Activates T3SS by Stabilizing the 5′ UTR of hrpG, the Master Regulator of hrp/hrc Genes, in Xanthomonas
Source: PLoS Pathog. 2014 Feb 27;10(2):e1003945. doi: 10.1371/journal.ppat.1003945 (PMC3937308; doi:10.1371/journal.ppat.1003945)
Supplement: Figure S2 — Graphical representation of the multiple sequence alignment and structural model of RsmA of Xanthomonas citri subsp. citri. A) Sequences of RsmA homologous proteins were used to generate this representation using the WebLogo server (http://weblogo.berkeley.edu/) [113], in which the height of the residue symbol indicates the degree of conservation and the numbers refer to residue positions in RsmA. RsmA secondary structure was predicted by Phyre (http://www.sbg.bio.ic.ac.uk/~phyre/) [50]. In of the predicted RsmA secondary structure, boxes indicate alpha-helices and arrows indicate β-strand motifs. B) The RsmA structure model was obtained using the Swiss-Model Repository program with the E. coli CsrA structure as a model [PDB: 1Y00, [48]]. The structure was generated using PyMOL [114]. The critical residues involved in RNA binding are highlighted in A) with stars and in B) with sticks in the RsmA three-dimensional structure model [33], [34]. (PDF) [file ppat.1003945.s002.pdf]

Figure S2.

A)

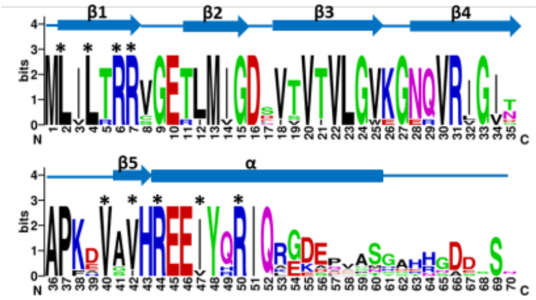

B)

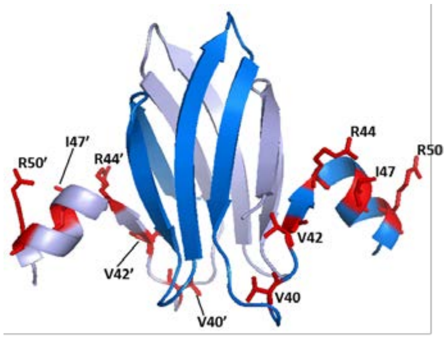

Figure S2. Graphical representation of the multiple sequence alignment and structural model of RsmA of *Xanthomonas citri* subsp. citri. A) Sequences of RsmA homologous proteins were used to generate this representation using the WebLogo server (http://weblogo.berkeley.edu/) [110], in which the height of the residue symbol indicates the degree of conservation and the numbers refer to residue positions in RsmA. RsmA secondary structure was predicted by Phyre (http://www.sbg.bio.ic.ac.uk/~phyre/) [49]. In the predicted XCC RsmA secondary structure, boxes indicate alpha-helices and arrows indicate β-strand motifs. **B)** The RsmA structure model was obtained using the Swiss-Model Repository program with the *E. coli* CsrA structure as a model [PDB: 1Y00, [47]]. The structure was generated using PyMOL [111]. The critical residues involved in RNA binding are highlighted in A) with stars and in B) with sticks in the RsmA three-dimensional structure model [32,33].
