# Supplementary material for: The Post-transcriptional Regulator rsmA/csrA Activates T3SS by Stabilizing the 5′ UTR of hrpG, the Master Regulator of hrp/hrc Genes, in Xanthomonas
Source: PLoS Pathog. 2014 Feb 27;10(2):e1003945. doi: 10.1371/journal.ppat.1003945 (PMC3937308; doi:10.1371/journal.ppat.1003945)
Supplement: Figure S3 — Induced expression of 6HisRsmA restores the production levels of endoglucanases in rsmA mutant of X. citri subsp. citri. A) Expression of 6HisrsmA upon induction with 0.25% L-arabinose was confirmed by Western-blot analysis with anti-6His antiserum. X. citri subsp. citri strains were growth in XVM2 medium at 28°C and OD600 nm 0.5, and after 4 hours of induction with 0.25% L-arabinose the cells were collect and centrifuged. Supernatants were discarded and cells were resuspended in Laemmli sample buffer. Proteins extracts were analyzed by SDS-PAGE, transferred to nitrocellulose membrane and incubated with anti-6His antiserum. E. coli cell extract expressing the recombinant protein 6HisRsmA was used as a positive control. B) Induced expression of 6HisRsmA restores the production levels of endoglucanases in rsmA mutant of X. citri subsp. citri. Endoglucanases activity assays were performed with supernatant of the strains growth in NB medium supplemented with 0.25% L-arabinose at 28°C and OD600 nm 1.0. Wt, XCC strain 306; pBRA, mutant rsmA harboring empty plasmid pBRA; pBRA6HisrsmA, rsmA mutant transformed with plasmid-borne 6HisrsmA under control of an arabinose induced promoter. (PDF) [file ppat.1003945.s003.pdf]

Figure S3.

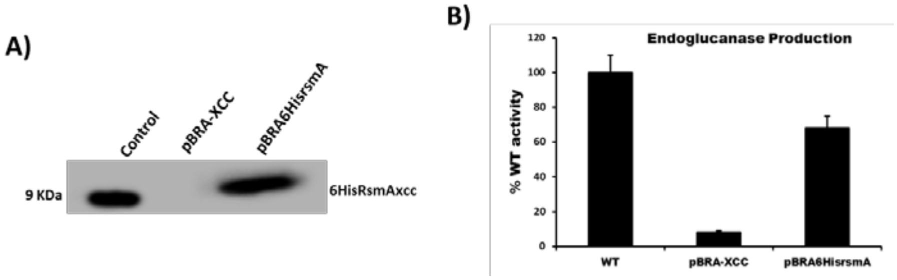

Figure S3. Induced expression of 6HisRsmA restores the production levels of endoglucanases in *rsmA* mutant of *X. citri* subsp. citri. A) Expression of 6HisrsmA upon induction with 0.25% L-arabinose was confirmed by Western-blot analysis with anti-6His antiserum. *X. citri* subsp. citri strains were growth in XVM2 medium at 28^0C and OD_{600nm} 0.5, and after 4 hours of induction with 0.25% L-arabinose the cells were collect and centrifuged. Supernatants were discarded and cells were resuspended in Laemmli sample buffer. Proteins extracts were analyzed by SDS-PAGE, transferred to nitrocellulose membrane and incubated with anti-6His antiserum. *E. coli* cell extract expressing the recombinant protein 6HisRsmA was used as a positive control. **B)** Induced expression of 6HisRsmA restores the production levels of endoglucanases in *rsmA* mutant of *X. citri* subsp. citri. Endoglucanases activity assays were performed with supernatant of the strains growth in NB medium supplemented with 0.25% L-arabinose at 28 ^{0}C and OD_{600nm} 1.0. (Slater et al. 2000; Ryan et al. 2010). Wt, XCC strain 306; pBRA, mutant *rsmA* harboring empty plasmid pBRA; pBRA6HisrsmA, *rsm*A mutant transformed with plasmid-borne 6HisrsmA under control of an arabinose induced promoter.
