# Supplementary material for: The Post-transcriptional Regulator rsmA/csrA Activates T3SS by Stabilizing the 5′ UTR of hrpG, the Master Regulator of hrp/hrc Genes, in Xanthomonas
Source: PLoS Pathog. 2014 Feb 27;10(2):e1003945. doi: 10.1371/journal.ppat.1003945 (PMC3937308; doi:10.1371/journal.ppat.1003945)
Supplement: Table S1 — Bacterial strains and plasmids used in this study. (PDF) [file ppat.1003945.s004.pdf]

Table S1. Bacterial strains and plasmids used in this study.

Strain or plasmid	Relevant characteristics	Reference
**Strains**		
Escherichia coli		
DH5α	F⁻ *rec*A (r_K–, m_K+) *hsd*R17 (rΔ(*lac*ZYA-*arg*F) Φ80d*lac*Z ΔM15	New England Biolabs
BL21 Star (DE3) pLysS	F⁻ ompT hsdSB (r_B- m_B-) gal dcm rne131 (DE3) pLysS (Cam^r)	Life Invitrogen
X. citri **subsp. citri** (XCC)		
306 (A)	Wild type, amp^r, rif^r	Da Silva *et al*., 2002.
Δ***rsmA***	In frame deletion of *rsmA* also named *csrA*	This study
Δ***hrpG***	*hrpG* mutant	Guo *et al*., 2011.
pBRA-XCC	Empty pBRA plasmid (Broad Range Arabinose inducible promoter) in the Δ*rsmA* background	This study
pBRArsmA	pBRA expressing 6His*rsmA* under control of arabinose inducible promoter in the Δ*rsmA* background	This study
Wt-*hrp**G***-6His	Wild-type strain carrying the construct *hrpG*-6His inserted in its chromosome by using an integrative plasmid pPM7g	This study
Δ***rsmA***-*hrp**G***-6His	Δ*rsmA* strain carrying the construct *hrpG*-6His inserted in its chromosome by using an integrative plasmid pPM7g	This study
Wt-*hrcQ*-Flag	Wild-type strain carrying the construct *hrcQ*-Flag inserted in its chromosome by using an integrative plasmid pPM7g	This study
Δ***rsmA***-*hrcQ*-Flag	Δ*rsmA* strain carrying the construct *hrcQ*-Flag inserted in its chromosome by using an integrative plasmid pPM7g	This study
**Plasmids**		
pGEM-T easy	PCR cloning vector, Ap^r	Promega
pGEMT-5UTRhrpG	5' untranslated region of *hrpG* cloned in pGEM-Teasy	This study
pGEMT-5UTRhrpD	5' untranslated region of *hrpD* operon cloned in pGEM-Teasy	This study
pGEMT-5UTRhrpE	5' untranslated region of *hrpE* operon cloned in pGEM-Teasy	This study
pNTPS138	Suicide vector for generation of gene knockouts, *sacB* and Km^r	M. R. Alley, unpublished
pNPTS-rsmA	pNPTS138 derivative for generation of *rsmA* knockout, *sacB* and Km^r	This study
pET28a(+)	*E. coli* expression vector, Km^r	Novagen
pET-6HisrsmA	*rsmA* cloned into pET28a$_{NdeI/HindIII}$	This study
pBRA	Vector for gene overexpression containing arabinose-inducible promoter P(BAD), Spc^r and Str^r	M. Marroquin, unpublished
pBRA6HisrsmA	pBRA derivative encoding 6HisRsmA	This study
pUFR047	Broad host range vector, Gm^r	De Feyter et al., 1993
pUFRrsmA	pUFR047 derivative encoding RsmA	This study
pUFRrsmA-flag	pUFR047 derivative encoding RsmA-Flag	This study
pUFRhrpG	pUFR047 derivative encoding HrpG	This study
pBI 121	Binary vector containing *gusA*, Km^r	Clontech
pLAFR3	Broad host range cloning vector, Tc^r	Huynh *et al*., 1989
pLAFRhrpG::gus	pLAFR3 expressing a HrpG-*gusA* fusion under control of *hrp*G native promoter	This study
pLAFRhrpX::gus	pLAFR3 expressing a HrpX-*gusA* fusion under control of *hrp*X native promoter	This study
pLAFRhrpA::gus	pLAFR3 expressing a HrcC-*gusA* fusion under control of *hrpA* native promoter	This study
pLAFRhrpB::gus	pLAFR3 expressing a HrpB1-*gusA* fusion under control of *hrpB* operon native promoter	This study
pLAFRhrpC::gus	pLAFR3 expressing a HrcU-*gusA* fusion under control of *hrpC* operon native promoter	This study
pLAFRhrpD::gus	pLAFR3 expressing a HrcQ-*gusA* fusion under control of *hrpD* operon native promoter	This study
pLAFRhrpE::gus	pLAFR3 expressing a HrpD5-*gusA* fusion under control of *hrpE* operon native promoter	This study
pBBR1-MCS-5	Broad host range vector, Gm^r	Kovach et al, 1995
pBBELac-hrpG6his	pBBR derivative encoding HrpG-6His under control of LAC promoter	This study
pBBRLac-hrpGD41N6his	pBBR derivative encoding HrpGD41N6His under control of LAC promoter	This study
pBBRLac-hrpGE44K6his	pBBR derivative encoding HrpGE44K6His under control of LAC promoter	This study
pBBRLac-hrpGD60N6his	pBBR derivative encoding HrpGD60N6His under control of LAC promoter	This study
pPM7g	Integrative plasmid containing a fragment *amy*106–912, Km^r	Martins *et al*., 2010
pPM7-hrpG6-His	pPM7g derivative encoding HrpG-6His under control of its native promoter	This study
pPM7-hrcQ-Flag	pPM7g derivative encoding HrcQ-Flag under control of *hrpD* operon native promoter	This study
pUC18-mini-Tn7T-LAC	Suicide delivery vector and mini-Tn7 elements, Ap^r and Gm^r	Choi *et al*., 2005
pTNS2	Helper vector	Choi *et al*., 2005
Tn7T-LAC-hrpG-GUS	pUC18-mini-Tn7T-LAC expressing a *hrp*G 5' UTR-*gus*A fusion under control of LAC promoter	This study
Tn7T-LAC-hrpB-GUS	pUC18-mini-Tn7T-LAC expressing a *hrp*B 5' UTR-*gus*A fusion under control of	This study

	LAC promoter	
Tn7T-LAC-hrpC-GUS	pUC18-mini-Tn7T-LAC expressing a *hrp*C 5’ UTR-*gus*A fusion under control of LAC promoter	This study
Tn7T-LAC-hrpD-GUS	pUC18-mini-Tn7T-LAC expressing a *hrp*D 5’ UTR-*gus*A fusion under control of LAC promoter	This study
Tn7T-LAC-hrpE-GUS	pUC18-mini-Tn7T-LAC expressing a *hrp*E 5’ UTR-*gus*A fusion under control of LAC promoter	This study

Ap^r, ampicillin-resistant; Gm^r, gentamycin-resistant; Km^r, kanamycin-resistant; Spc^r, spectinomycin-resistance; Str^r, streptomycin-resistance; Tc^r, tetracycline-resistance.
