# Supplementary material for: The Post-transcriptional Regulator rsmA/csrA Activates T3SS by Stabilizing the 5′ UTR of hrpG, the Master Regulator of hrp/hrc Genes, in Xanthomonas
Source: PLoS Pathog. 2014 Feb 27;10(2):e1003945. doi: 10.1371/journal.ppat.1003945 (PMC3937308; doi:10.1371/journal.ppat.1003945)
Supplement: Table S2 — Comparison of expression of the genes regulated by rsmA in Xanthomonas citri subsp. citri. using microarray. (PDF) [file ppat.1003945.s005.pdf]

Table S2. Comparison of expression of the genes regulated by *rsmA* in *Xanthomonas citri* subsp. citri. using microarray.

Locus tag	Gene name	Log$_2$ ratio of fold change (Δ*rsmA*/WT)[a]	Log$_2$ ratio of fold change (pBRArsmA/pBRA)[b]	Product description
		**Down-regulated**	**Up-regulated**	
XAC0035	-	-1.01	2.37	hypothetical
XAC0076	*avrBS2*	-1.00	2.17	putative type 3 secretion effector
XAC0104	-	-1.09	2.37	metalloprotease
XAC0105	-	-1.10	-	hypothetical
XAC0155	-	-1.13	2.32	Trehalose synthase
XAC0224	*poxB*	-1.05	1.98	pyruvate dehydrogenase
XAC0225	*ntrB*	-1.15	2.76	histidine kinase
XAC0226	*ntrC*	-1.20	3.63	response regulator
XAC0239	-	-1.40	-	hypothetical
XAC0277	*xopR*	-2.10	2.8	putative type 3 secretion effector
XAC0286	*xopE1*	-1.80	2.68	putative type 3 secretion effector
XAC0393	*hpaF (xopAE)*	-1.41	2.58	type 3 secretion effector
XAC0394	*hrpF*	-1.23	2.79	type 3 secretion protein HrpF
XAC0396	*hpaB*	-1.13	3.31	type 3 secretion protein HpaB
XAC397	*hrpE*	-1.44	2.65	type 3 secretion protein HrpE
XAC0398	*hrpD6*	-1.30	2.87	type 3 secretion protein HrpD6
XAC0399	*hrpD5*	-1.56	3.50	type 3 secretion protein HrpD5
XAC0400	*hpaA*	-1.25	3.58	type 3 secretion protein HpaA
XAC0401	*hrcS*	-1.31	3.62	type 3 secretion protein HrcS
XAC0402	*hrcR*	-1.41	3.53	type 3 secretion protein HrcR
XAC0403	*hrcQ*	-1.73	3.98	type 3 secretion protein HrcQ
XAC0404	*hpaP*	-1.10	3.37	type 3 secretion protein HpaP
XAC0405	*hrcV*	-1.26	3.35	type 3 secretion protein HrcV
XAC0406	*hrcU*	-1.40	3.78	type 3 secretion protein HrcU
XAC0407	*hrpB1*	-1.37	3.48	type 3 secretion protein HrpB1
XAC0408	*hrpB2*	-1.32	3.92	type 3 secretion protein HrpB2
XAC0409	*hrcJ*	-1.82	3.91	type 3 secretion protein HrcJ
XAC0410	*hrpB4*	-1.75	4.05	type 3 secretion protein HrpB4
XAC0411	*hrpB5*	-1.46	4.12	type 3 secretion protein HrpB5
XAC0412	*hrcN*	-1.23	3.78	type 3 secretion protein HrcN
XAC0413	*hrpB7*	-1.17	4.02	type 3 secretion protein HrpB7
XAC0414	*hrcT*	-1.00	3.96	type 3 secretion protein HrcT
XAC0415	*hrcC*	-1.06	2.51	type 3 secretion protein HrcC
XAC0416	*hpa1*	-1.72	3.28	type 3 secretion protein Hpa1
XAC0417	*Hpa2*	-1.13	2.53	type 3 secretion protein Hpa2
XAC0435	*virK*	-1.08	2.15	VirK
XAC0494	-	-1.10	-	histidine kinase
XAC0501	-	-1.24	2.96	hypothetical
XAC0543	*xopX*	-1.15	2.52	hypothetical
XAC0601	*xopV*	-	2.01	putative type 3 secretion effector
XAC0637	*hslV*	-1.18	1.67	ATP-dependent protease peptidase subunit
XAC0638	*hslU*	-1.29	1.73	ATP-dependent protease ATP-binding subunit
XAC0644	-	-1.12	-	diguanylate cyclase
XAC0754	*xopI*	-1.28	2.87	putative type 3 secretion effector
XAC0878	*pcaH*	-1.05	3.33	protocatechuate 4.5-dioxygenase subunit beta
XAC0879	*ligA*	-1.00	3.49	protocatechuate 4.5-dioxygenase subunit alpha
XAC0880	*pcaQ*	-1.12	3.01	transcriptional regulator
XAC0916	-	-1.09	2.37	hydrolase
XAC1124	-	-1.13	2.52	hypothetical

XAC1171	*stkxac1*	-1.13	2.37	serine/threonine kinase
XAC1172	-	-1.32	3.40	hypothetical
XAC1208	*xopP*	-1.07	3.43	putative type 3 secretion effector
XAC1211	*katE*	-1.04	2.54	catalase
XAC1242	*pthX*	-1.06	-	pathogenicity-related protein
XAC1265	*hrpG*	-1.23	1.32	OmpR transcriptional regulator
XAC1266	*hrpX*	-1.18	1.41	AraC transcriptional regulator
XAC1319	*algU*	-1.05	2.81	sigma factor RpoE
XAC1354	-	-1.05	-	hypothetical
XAC1525	*tyrA*	-1.36	2.05	prephenate dehydrogenase
XAC1682	*rpoE*	-	2.68	Sigma factor RpoE
XAC1743	*csrA/rsmA*	-5.38	4.62	post-transcriptional regulator
XAC2053	*tex*	-1.08	2.89	transcription-related protein
XAC2164	-	-1.73	2.03	hypothetical
XAC2165	-	-1.46	2.15	isochorismatase
XAC2166	-	-1.58	1.85	AraC transcriptional regulator
XAC2399	*htpX*	-1.42	1.96	heat shock protein HtpX
XAC2528	*hptG*	-1.38	-	heat shock protein HtpG
XAC2786	*xopN*	-1.51	3.61	type 3 secretion effector
XAC2853	-	-1.25	1.63	cysteine protease
XAC2922	*hrpW*	-1.21	1.88	type 3 secretion protein HrpW
XAC2946	-	-1.80	-	hypothetical
XAC3085	*xopK*	-1.47	3.89	putative type 3 secretion effector
XAC3090	*xopL*	-	3.01	putative type 3 secretion effector
XAC3230	*xopAI*	-1.10	1.51	putative type 3 secretion effector
XAC3647	*pheA*	-1.44	3.29	Chorismate mutase
XAC3666	*xopAK*	-1.06	2.41	putative type 3 secretion effector
XAC3683	-	-1.00	1.97	histidine kinase
XAC3685	-	-1.27	2.41	hypothetical
XAC3878	-	-1.22	-	disulphide-isomerase
XAC4213	*xopAD*	-1.31	3.37	putative type 3 secretion effector
XAC4235	-	-1.36	-	hypothetical
XAC4269	-	-1.20	2.53	hypothetical
XAC4327	*uahA*	-1.17	3.69	allophanate hydrolase
XAC4333	*xopQ*	-1.18	2.64	putative type 3 secretion effector
XACb0011	*xopE3*	-1.08	2.12	putative type 3 secretion effector
		**Upregulated**	**Downregulated**	
XAC0028	*egl*	3.05	-5.35	cellulase
XAC0298	-	1.10	-	hypothetical
XAC0346	*celA*	2.11	-4.6	cellulase
XAC0466	-	1.64	-3.70	lytic enzyme
XAC0467	-	1.12	-3.62	hypothetical
XAC0545	*aroG*	1.42	-2.01	phospho-2-dehydro-3-deoxyheptonate aldolase
XAC0604	*treA*	2.26	-1.53	trehalase
XAC0827	*nrtB*	1.65	-4.31	permease
XAC0828	*nrtCD*	1.82	-4.46	ABC transporter ATP-binding component
XAC0829	-	2.23	-5.11	ABC transporter substrate binding protein
XAC0830	*tauD*	2.08	-5.15	taurine dioxygenase
XAC0851	*slfA*	1.25	-5.01	NADH-dependent FMN reductase
XAC0868	-	1.86	-4.55	hypothetical
XAC1034	-	-	-4.28	peptidyl-Asp metalloendopeptidase
XAC1145	-	1.12	-	hypothetical
XAC1200	-	1.05	-3.95	prolyl oligopeptidase family protein
XAC1264	-	1.20	-1.52	hypothetical
XAC1284	-	-	-2.25	response regulator protein
XAC1328	-	1.98	-4.34	response regulator
XAC1397	-	1.54	-	padR transcriptional regulator
XAC1426	*ecpD*	2.85	-3.94	pili assembly chaperone

XAC1427	*pru*	2.14	-5.21	protein U
XAC1495	*cspA*	-	-3.94	major cold shock protein
XAC1492	-	2.16	-5.03	hypothetical
XAC1573	*phoU*	2.65	-2.05	phosphate regulon transcriptional regulator
XAC1778	-	1.44	-4.28	histidine kinase
XAC1779	-	1.18	-3.68	hypothetical
XAC1877	*rpfG*	2.26	-1.50	phosphodiesterase
XAC1878	*rpfC*	1.07	-	histidine kinase
XAC1906	*cheW*	1.78	-2.94	chemotaxis protein
XAC1907	*parA*	2.20	-3.46	chromosome 3ortioning protein
XAC1909	*motD*	1.72	-	flagellar motor protein MotD
XAC1930	*cheA*	1.42	-4.03	chemotaxis related protein
XAC1931	*cheZ*	2.25	-5.21	chemotaxis related protein
XAC1932	*cheY*	2.44	-5.37	chemotaxis protein
XAC1933	*fliA*	2.33	-5.12	RNA polymerase sigma factor
XAC1934	*fleN*	2.72	-5.35	flagellar biosynthesis switch protein
XAC1935	*flhF*	2.61	-5.28	flagellar biosynthesis regulator
XAC1936	*flhA*	2.20	-3.84	flagellar biosynthesis protein
XAC1937	*flhB*	1.80	-2.32	flagellar biosynthesis protein
XAC1941	*fliR*	2.18	-1.71	flagellar biosynthesis protein
XAC1942	*fliQ*	1.78	-1.81	flagellar biosynthesis protein
XAC1944	*fliP*	2.40	-2.01	flagellar biosynthesis protein
XAC1945	*fliO*	1.77	-3.82	flagellar protein
XAC1946	*fliN*	2.29	-3.79	flagellar protein
XAC1947	*fliM*	2.02	-3.48	flagellar protein
XAC1948	*fliL*	1.83	-3.04	flagellar protein
XAC1949	*fliK*	1.71	-3.32	flagellar protein
XAC1954	*fliF*	1.56	-2.84	flagellar MS-ring protein
XAC1955	*fliE*	1.69	-4.12	flagellar protein
XAC1965	*acp*	1.82	-1.83	acyl carrier protein
XAC1966	*vioA*	1.42	-	nucleotide sugar transaminase
XAC1974	*fliD*	1.44	-4.07	flagellar protein
XAC1975	*fliC*	1.51	-5.21	flagellin
XAC1976	*flgL*	1.48	-5.85	flagellar hook-associated protein
XAC1980	*flgH*	1.42	-5.48	flagellar basal body L-ring protein
XAC1983	*flgE*	1.57	-5.01	flagellar hook protein FlgE
XAC1986	*FlgB*	1.62	-5.30	flagellar basal body rod protein FlgB
XAC1990	-	2.19	-2.64	hypothetical
XAC2135	-	1.38	-2.01	hypothetical
XAC2151	*yapH*	1.18	-5.43	Filamentous hemagglutinin-related protein
XAC2250	-	1.26	-4.32	hypothetical
XAC2443	-	1.43	-2.98	hypothetical
XAC2463	-	1.24	-3.18	hypothetical
XAC2529	*rhsD*	1.72	-5.43	RhsD
XAC2608	*virB6*	2.47	-3.31	VirB6
XAC2609	-	2.81	-4.01	hypothetical
XAC2610	-	2.23	-3.92	hypothetical
XAC2614	*virB4*	1.52	-2.18	VirB4
XAC2616	*virB2*	2.44	-4.71	VirB2
XAC2618	*virB11*	2.39	-4.73	VirB11
XAC2619	*virB10*	1.98	-5.23	VirB10
XAC2663	-	1.07	-3.37	transposase
XAC2664	*pilE*	1.05	-4.13	PilE protein
XAC2774	-	1.31	-5.49	TonB
XAC2885	-	1.86	-1.57	phospholipase A1
XAC2897	-	1.97	-	diguanylate cyclase
XAC2992	-	1.17	-5.32	endoproteinase Arg-C
XAC3031	-	1.53	-3.21	histidine kinase
XAC3241	*fimA*	1.31	-5.61	FimA

XAC3262	-	1.08	-2.86	hypothetical
XAC3267	-	1.12	-2.18	hypothetical
XAC3279	-	2.39	-5.71	hypothetical
XAC3340	*cysG*	1.78	-4.72	siroheme synthase
XAC3380	-	1.12	-4.76	hypothetical
XAC3533	-	1.83	-2.83	hypothetical
XAC3634	-	1.61	-3.63	hypothetical
XAC3636	-	2.10	-2.71	hypothetical
XAC4011	-	2.42	-3.19	hypothetical
XAC4026	-	2.01	-4.31	hypothetical
XACa0013	-	1.08	-2.06	hypothetical

[a]The Log_2-fold change of each gene was derived from the comparasion of *rsmA* mutant versus wild-type ($p<0.05$; Log2>=1). [b] The Log_2-fold change of each gene was derived from the comparasion of the Δ*rsmA* mutant carrying the construct pBRArsmA and empty plasmid pBRA. Transcriptome analyses were performed with four independent biological samples for each condition.
