# Supplementary material for: The Post-transcriptional Regulator rsmA/csrA Activates T3SS by Stabilizing the 5′ UTR of hrpG, the Master Regulator of hrp/hrc Genes, in Xanthomonas
Source: PLoS Pathog. 2014 Feb 27;10(2):e1003945. doi: 10.1371/journal.ppat.1003945 (PMC3937308; doi:10.1371/journal.ppat.1003945)
Supplement: Table S3 — Oligonucleotides primers used in this study. (PDF) [file ppat.1003945.s006.pdf]

Table S3. Oligonucleotides primers used in this study.

Name	Sequence (5' to 3')
For mutant construction	
F1ΔrsmA	CACCTGAATTCGACTGCGCTTTGACTTCTCG
R1ΔrsmA	CGGCGAAGCTTGAGGATCAACATTTTGCAAATC
F2ΔrsmA	ATCACGGCGACGATTCTTCGAAGCTTTGATCG
R2ΔrsmA	GGTTTTGGAATTCTTGATCCTTACTCCTTGATGGTC
For Complementation	
F-rsmAc	CACCTGAATTCGACTGCGCTTTGACTTCTCG
R-rsmAc	CTAACGTAAACCCTTGAATTCAACGATCAATTGGAAG
F1-rsmAflag	CTGGATCCCTTGTCGCTGGCGTGAACG
R1-rsmAflag	CTTGTAATCATTGGAAGAATCGTCGCCGTG
R2-rsmAflag	GAACTCGAGTCACTTGTCATCATCATCCTTGTAATCATT
F-hrpG	CAGGATCCGCGATGCCCGATGACTGTACG
R-hrpG	TATAAGCTTCGCAGATCGGCAGCGGTG
Fwconst-hrpG	ATAGGTACCATGCGTGGCTTGTACGCCGAC
R1-hrpG6His	GATGGTGATGGCAGGCGGCTGCGTGATGTGC
R2-hrpG6His	CCCTCGAGTCAGTGATGGTGATGGTGATGGCAGG
F-hrcQ	CTTAGGTACCGAGTTCTGCAACGCCACGCCAGTG
R1-hrpQFlag	GTAATCGGCATCTGCATGCGTGCTCTCCGACAT
R2-hrpQFlag	GTACTCGAGTTACTTATCGTCGTCATCCTTGTAATCGGCATC
For 5' RACE	
hrpGSP1	GTCTGCGCAATCGAAACGTCCAAG
hrpGSP2	GAAGATCAGCAGCTCGCACGGC
hrpGSP3	GCTGGCGTTGACCTGCGAGACC
hrpXSP1	CTGGCACCGAACAAACTGACCTCATC
hrpXSP2	CGCGTCGCTGTTGGAGATGTTGC
hrpXSP3	GGCCAAGCACCAACCAGCATCC
hrpBSP1	CGTGTCCAGCGCCTTCAGCTCTGGCCG
hrpBSP2	AGTACTCGGACTGCGGCCAGGACC
hrpCSP1	TGGTCGGAGAAGTAGTCGCCGGCCG
hrpCSP2	CATGACCATTAGCGCGCCAAAGAGC
hrpDSP1	GTTCAGTAGCAGCGCCAGGCCGTCG
hrpDSP2	CGGTGTCCAACTGCAGGCGCAGGC
hrpESP1	GCTAGCATGGCACCCACCGCAGTAC
hrpESP2	GCAAGACTTTGCGCCTGGACGTGC
hrpFSP1	GACGGCAGATCGGAGTCATCCTTGGA
hrpFSP2	GAATCCGACCCCAACAGCTCCGAC
For translational and transcriptional fusions	
gusA-Fw	ATGTTACGTCCTGTAGAAACCCCAACCCGTG
gusA-R	GTAAGCTTCATTGTTTGCCTCCCTGCTGCG
hrpG-Fw	CTTGGTACCTCACTCTGTCAAACATCCATGTGATG
hrpG-R	GGGTTTCTACAGGACGTAACATGGGAGAGTGGTCGTTCATTTAGG
hrpX-Fw	GTATGGTACCGTCCGCCGAGGTCTAGACCAACC
hrpX-R	GTTGGGGTTTCTACAGGACGTAACCAAGCACCAACCAGCATCCTGC
hrpA-Fw	GATTGGTACCTGGTGGATCTAGCGATCGGCCTGG
hrpA-R	GTTGGGGTTTCTACAGGACGTAACATCAGGACGGCGGCCAATGGAG
hrpB-Fw	GATTGGTACCGACATTGCCCTCTCCTTCCGCG
hrpB-R	GTTGGGGTTTCTACAGGACGTAACAGTACTCGGACTGCGGCCAGGAC
hrpC-Fw	GTATGGTACCTGAATCTTGTCATGGGTGAGTCCGACC
hrpC-R	GTTGGGGTTTCTACAGGACGTAACATTAGCGCGCCAAAGAGCAC
hrpD-Fw	CTTAGGTACCGAGTTCTGCAACGCCACGCCAGTG
hrpD-R	GTTGGGGTTTCTACAGGACGTAAGATGCGACGGCGATGCGTGGCGAAG
hrpE-Fw	GTCGAATTCGAGCTGATGCCGTCAGAGTTGG
hrpE-R	GTTGGGGTTTCTACAGGACGTAAGGCATCGAACATAGTGGACTCTC
hrpG5UTR-F	CGGGTCCCGGCAATGGTACATACC
hrpB5UTR-F	TGCTTCCGAAAGGATCCGGCC
hrpC5UTR-F	CTGTCAAGCGGATCGTTCCACCACAGCT
hrpD5UR-F	GAAGGCTGCGTCTGGAGGCTGCACT
hrpE5UTR-F	AGGTCGTTACACCCTGGGGAGCGAT
For expression of rsmA in pET28a and pBRA	
F-pETrsmA	GAGATTTGCAAACATATGTTGATCCTCACTCGCCG
R-pETrsmA	CTAACGTAAACCCTTGAATTCAACGATCAATTGGAAG

For site-direct mutagenesis	
hrpGD41N-F	AATGTCTCGACGTTTTCTAATGAACTGGAGCTTTTGCGTACGCTGC GGC
hrpGD41N-R	GCCGCAGCGTACGCAAAAGCTCCAGTTCATTAGAAAACGTCGAGA CATT
hrpGE44K-F	ACGTTTTCCGATGAACTGAAGCTTTTGCGTACGCT
hrpGE44K-R	AGCGTACGCAAAAGCTTCAGTTCATCGGAAAACGT
hrpGD60N-F	CTGATCTTCAATGCCAGCTATGTCGCCGCCGATGACAGTT
hrpGD60N-R	CTGGCATTGAAGATCAGCAGCTCGCACGGCGAATG
hrpGBS1-TTA-F	TCGTCCAGCTCCGCTTTACTCTCCCCCGCCAAC
hrpGBS1-TTA-R	GTTGGCGGGGGAGAGTAAAGCGGAGCTGGACGA
hrpGBS2-TTA-F	GATGCAGCGATCTTTGAAGATTTATTTTGTCCTGGCATTGG
hrpGBS2-TTA-R	CCAATGCCAGGACAAAATAAATCTTCAAAGATCGCTGCATC
hrpGΔBS2-F	AGCGATAAGCTTCATTGGCCGACCAGCACCG
hrpGΔBS2-R	AAGCTTATCGCTGCATCGTCGGACCAGTCGG
For in vitro transcription	
hrpG5UTR-F	CGGGTCCCGGCAATGGTACATACC
hrpG5UTR-R	CGTTGGGGGGAGAGTGGTCGTCAT
hrpD5UTR-F	GAAGGCTGCGTCTGGAGGCTGCACT
hrpD5UTR-R	ACCCTGCTCAGCTGTGCGCGCTC
hrpE5UTR-F	AGGTCGTTACACCCTGGGGAGCGAT
hrpE5UTR-R	TGCAGAAAGCGCTAGCATGGCACC
hrpG5UTR-T7-F	TAATACGACTCACTATAGGGGGTCCCGGCAATGGTACATAC
hrpG5UTR-T7-R	GGAGAGTGGTCGTCATTTAGGCGGCCTTCG
hrpD5UTR-T7-F	TAATACGACTCACTATAGGGAAGGCTGCGTCTGGAGGCTG
hrpD5UTR-T7-R	TGATACCCTGCTCAGCTGTGCGCGCTCGG
hrpG5UTR-R-81	AAGCTTATCGCTGCATCGTCGGACCAGTCGG
hrpG5UTR-T7-F-54	TAATACGACTCACTATAGGGCAACCGACTGGTCCGACGATG
For qRT-PCR	
hrpE $_{XAC0397}$-F	TCGTTGATCAGTGACATGAACGCC
hrpE $_{XAC0397}$-R	GATGAACTTGTTCAGCGCCACGTT
hrpD6 $_{XAC0398}$-F	TCGATGCCATGACCGATACG
hrpD6 $_{XAC0398}$-R	TTGAAGTCGTTGCGTGAGGT
hrpD5 $_{XAC0399}$-F	GTCCGTTACAACGGCAATGG
hrpD5 $_{XAC0399}$-R	TTCGACATAATGCAGGCCGT
hrcS $_{XAC0401}$-F	CCACGACGATCTAGTGCGAT
hrcS $_{XAC0401}$-R	ACATCACCGCCTGGATGAAG
hrcQ $_{XAC0403}$-F	CAGCACGATACGTTTGAGCC
hrcQ $_{XAC0403}$-R	TTCGCCCACTGTCACTAACC
hrcU $_{XAC0406}$-F	TGATCATTGGCCTTGCCTACCAGA
hrcU $_{XAC0406}$-R	TTGCTCATTCGTTTGTCGCGGATG
hrpB1 $_{XAC0407}$-F	ACCCATGACAAGATTCAGGACGCT
hrpB1 $_{XAC0407}$-R	CTTCCACGTAATTACCGCGCTTGA
hrpB2 $_{XAC0408}$-F	CCGGCACCAGTATTACCTCC
hrpB2 $_{XAC0408}$-R	ATCGATATGCTCGGCGATGG
hrcC $_{XAC0415}$-F	ATACGTCGCCGACAACAAGGATCT
hrcC $_{XAC0415}$-R	CGGAGATGTTTCGAATTTGCCGCT
hpa1 $_{XAC0416}$-F	ATTCTTTGAACACACAGCTCGGCG
hpa1 $_{XAC0416}$-R	TCGGCATTGTTGCTCTGCTGAA
hpa2 $_{XAC0417}$-F	ACAACAACGGCACGGTTGATTACG
hpa2 $_{XAC0417}$-R	ACTGCAGCCCAGGTATTTCCGTAT
hrpG $_{XAC1265}$-F	ATCGTGCTTGGACGTTTCGATTGC
hrpG $_{XAC1265}$-R	ATTGAAAGGCAGCGCAAGGACTTC
hrpX $_{XAC1266}$-F	AGCGATCTCTGCGTTGTCCTAC
hrpX $_{XAC1266}$-R	ATACGCATCTTCGGCCTCTTCCTGA
16S-F	CGCTTTCGTGCCTCAGTGTCAGTGTTGG
16S-R	GGCGTAAAGCGTGCGTAGGTGGTGGTT

Restriction enzyme sites used in this study are underline in the primer sequences.
