# Supplementary material for: The Post-transcriptional Regulator rsmA/csrA Activates T3SS by Stabilizing the 5′ UTR of hrpG, the Master Regulator of hrp/hrc Genes, in Xanthomonas
Source: PLoS Pathog. 2014 Feb 27;10(2):e1003945. doi: 10.1371/journal.ppat.1003945 (PMC3937308; doi:10.1371/journal.ppat.1003945)
Supplement: Table S4 — RNA probes encoding 5′-UTR of hrp genes and control used in this study. (PDF) [file ppat.1003945.s007.pdf]

Table S4. RNA probes encoding 5’-UTR of *hrp* genes and control used in this study.

Name	Sequence (5’ to 3’)	Reference
**Control R9-43**	GGGAAUUCAACUCCAUCUAGGCACAAGGAUGUGCCAUAGUACUCAAGCU	Dubey *et al*., 2005
**hrpG1**	AUUCGUCCAGCUCCGCUGGACUCUC	This study
**hrpG2**	ACGAUGCAGCGAUCUUUGAAGAUGGAUUUUGUCCUGGCAUUG	This study
**hrpX**	GUUAGGCGCCUGUUGUCUUUUGCUCCGCCCCCCAAAGAGAGAGAC	This study
**hrpB**	GUGUACGGUCCGCAUGUCAUUAAAACGAGGUGCAGUGUG	This study
**hrpC**	GCGCAGCGAUCGCGGAAGGAGAGGGCAAUG	This study
**hrpF**	GCCCAUCGCGCAGAUUGGCAACAUACGGCUACACCGCAUG	This study
